# Amino Silane High Positive Charge Layers: A Stable and Durable Alternative Based on Electrostatic Interactions for Titanium Applications, Combining Antimicrobial and Biological Properties

**DOI:** 10.1021/acsabm.5c00684

**Published:** 2025-09-23

**Authors:** João Pedro dos S. Silva, Mariana Mireski, Irene Mallor-Solís, Maria Helena Rossy Borges, Rodolfo Debone Piazza, Rodrigo Fernando Costa Marques, Nilson Cruz, Elidiane C. Rangel, Carlos A. Fortulan, José H. D. da Silva, Jean Geringer, Conrado Aparicio, Valentim A. R. Barão

**Affiliations:** † Department of Prosthodontics and Periodontology, Piracicaba Dental School, 28132 Universidade Estadual de Campinas (UNICAMP). Av. Limeira, 901, Piracicaba, São Paulo 13414-903, Brazil; ‡ Mines Saint-Etienne, 28133 Université de Lyon, Université Jean Monnet, INSERM, U 1059 Sainbiose, Centre CIS, Department BioMat, F-42023 Saint-Etienne, France; § Bioinspired Oral Biomaterials and Interfaces—BOBI, Department of Materials Science and Engineering, 16767 Universitat Politècnica de Catalunya (UPC)-Barcelona Tech, Barcelona 08019, Spain; ∥ Laboratory of Magnetic Materials and Colloids, Department of Analytical, Physico-Chemistry and Inorganic Chemistry, Institute of Chemistry, 28108 São Paulo State University (UNESP), Araraquara, SP 14800-060, Brazil; ⊥ Laboratory of Technological Plasmas, Institute of Science and Technology, São Paulo State University (UNESP), Av. Três de Março, 511, Sorocaba, São Paulo 18087-180, Brazil; # Department of Mechanical Engineering, University of São Paulo (USP), Trabalhador São Carlense, 400, São Carlos, São Paulo 13566-590, Brazil; ∇ Department of Physics, School of Sciences, São Paulo State University (UNESP), Av. Eng. Luís Edmundo C. Coube, 14-01, Bauru, São Paulo 17033-360, Brazil; ○ Catalan Institute for Research and Advanced Studies (ICREA), Barcelona 08010, Spain; ◆ Institute for Bioengineering of Catalonia (IBEC), Barcelona 08028, Spain

**Keywords:** titanium, cationic coating, electrostatic Interactions, biofilms, cell proliferation

## Abstract

Cationic coatings on titanium surfaces are a promising approach for dental and biomedical implants due to their low-cost antimicrobial effect and no need for antibiotics. These coatings are applied on hydroxylated (−OH) surfaces using silanes, such as 3-aminopropyltriethoxysilane (APTES). However, it is unclear whether the concentration of this organofunctional compound affects surface charge or potential toxicity. This study investigated how different concentrations of APTES in cationic coatings on titanium samples influence electrostatic behavior and interactions with bacteria and mesenchymal stem cells (MSCs). Titanium discs served as controls (Ti group) and were first treated by plasma electrolytic oxidation (PEO) to generate −OH groups (PEO group). Subsequently, APTES was applied at 83.8, 167.6, and 251.4 mM, forming PEO+APTES0.3, PEO+APTES0.6, and PEO+APTES0.9 groups, respectively. Surfaces were characterized by scanning electron microscopy (SEM), energy-dispersive X-ray spectroscopy (EDS), X-ray photoelectron spectroscopy (XPS), Fourier-transform infrared spectroscopy (FTIR), X-ray diffraction (XRD), contact angle, Zeta potential, and profilometry. Microbiological assays assessed initial bacterial adhesion (1 h) and biofilm formation (24 h) using *Staphylococcus aureus* and *Escherichia coli*. Cell metabolism was assessed on days 1, 3, and 8, while cell viability was assessed on days 1 and 3 using mesenchymal stem cells. PEO-treated surfaces showed porous morphology, and silanization increased roughness and shifted surfaces toward hydrophobicity. Amines and surface charge changes were confirmed by XPS and Zeta potential. Increasing APTES concentration did not proportionally increase cation number. Crystalline hydroxyapatite oxides were identified following the electrochemical process. SEM, EDS, and FTIR confirmed treatment stability after 28 days of immersion, while tribological tests indicated improved performance for PEO-treated groups. Cationic coatings reduced bacterial adhesion by up to 65%, decreased biofilm Log10 values, and increased dead bacteria proportion. Biocompatibility was confirmed by metabolism and cell viability tests, with the group with lower APTES concentration showing the best performance on day 8, with an 80% higher cell metabolism than day 1. On the other hand, higher concentrations of APTES resulted in reduced cell metabolism. These findings indicate, for the first time, that APTES concentration does not affect electrostatic properties but that lower concentrations are required for cytocompatible cationic coatings.

## Graphical abstract

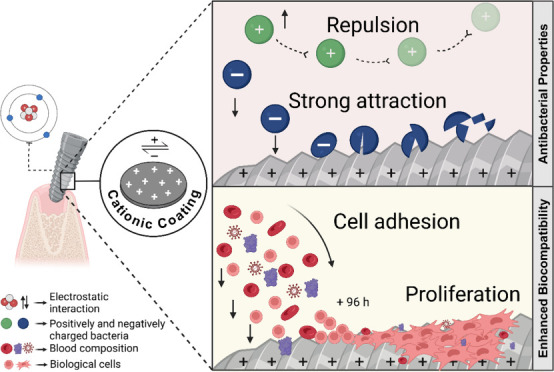

## 1 Introduction

Implants made from titanium and its alloys provide excellent options for oral and orthopedic rehabilitations.^1–3^ In recent years, the number of implant treatments has significantly increased, improving the patients’ quality of life.^4^ Nevertheless, the cost of these treatments is still high. For instance, in the United States, the investment for complex cases, such as hip or knee prostheses, can exceed USD 80,000.^5^ For dental implants, the costs are around USD 5,000, including the implant and the single prosthetic crown.^6^ Beyond the financial burden, some treatments face complications related to microbiological issues, including mucositis and/or peri-implantitis, often linked to poor hygiene or susceptibility to biofilm formation, particularly in chronic or immunosuppressed patients.^7,8^ Mucositis consists of an inflammatory process caused by the accumulation of biofilm that disrupts homeostasis at the implant-mucosa interface.^9^ The histopathological and clinical conditions of mucositis can potentially progress to peri-implantitis, characterized by the progressive loss of supporting bone.^10^ Despite the well-established etiology, there is still no consensus on the optimal clinical protocol to treat infections on dental implants. To overcome this challenge, researchers have been exploring different techniques, materials, and mechanisms aimed at treating and controlling peri-implant infections.^11–13^ Simultaneously, these approaches seek to enhance the antimicrobial effect while promoting an improved healing response.

Recently, developed a cationic antimicrobial coating characterized by a high positive charge.^12^ The mechanism of action for this type of coating is based on electrostatic interactions, where the positively charged coating attracts negatively charged microorganisms, with a consequent disruption of the microorganisms’ cell membranes.^12^ Additionally, when the bacterial cell shares the same charge as the coating, electrostatic repulsion occurs, reducing initial adhesion and controlling bacterial proliferation.^14^ Cationic coatings offer several advantages over other antimicrobial approaches. Besides not promoting bacterial resistance—a common issue with antibiotic treatments—these coatings tend to be stable and cost-effective to produce.^12^

To produce cationic coatings, the use of industrial silanes has become a widely accessible route. However, precautions must be taken when incorporating these chemical agents, as high concentrations can lead to cell toxicity. The failure in biocompatibility is often attributed to the byproducts generated during the hydrolysis of silanes, particularly the release of residual ethanol and the formation of silanol groups (Si–OH).^15–17^ Furthermore, byproducts can exacerbate inflammatory processes. Therefore, a thorough understanding of the mechanisms underlying this treatment is essential to ensure its effective application in biomedical titanium implants. Among the various silanes available, 3-aminopropyltriethoxysilane (APTES) stands out for its alkyl chain, which facilitates the formation of a stable anchor for chemical reactions, by covalent bonding with the alkalinized surface.^14^ The alkyl tail of the APTES molecule also imparts hydrophobic properties to the silanized surface, which hinders water interactions with the coating and thus, may prevent degradation of the coating by hydrolyzation of silanols.^14^ Furthermore, the presence of amine groups allows the formation of chemical bonds with other functional groups, improving the cohesion of atomic bonds and contributing to the coating’s durability. A further advantage of the amine group is its ability to accelerate blood flow and increase oxygen transport to the injured site, promoting a more efficient healing response.^18^ Additionally, the silanol group, upon hydrolysis, forms siloxane bonds (Si–O–Si) that enhance adhesion, thermal stability, chemical resistance and biocompatibility of the material.^19^

Considering the importance of surface treatments in the biomedical field and their impact on the global economy, these treatments must be effective, easy to perform, reproducible, and acceptable to the patient. In this context, the Plasma Electrolytic Oxidation (PEO) technique emerges as an excellent route for enabling cationic coatings. Several studies have shown the advantages of PEO, mainly its ability to standardize surfaces—a crucial for ensuring treatment reproducibility.^20,21^ Among its benefits, PEO can modify surface topography by forming volcanic pores that favor mechanical interlocking, and enable the incorporation of bioactive elements and functional groups.^12^ The incorporation of bioactive elements, such as calcium (Ca) and phosphorus (P), plays a fundamental role in improving cellular activity and fostering a more efficient healing response.^22^ The sodium hydroxide in the electrolytic solution creates hydroxyl groups (−OH), which bind to the silanol groups of the APTES, forming the cationic coatings.^12^ However, regardless of the method used to obtain these surfaces, no studies in the literature have explored the effects of varying silane concentrations or the associated risks. Silane plays a crucial role in increasing surface charge, but it remains unclear whether higher concentrations could further intensify this charge, altering electrostatic interactions with microbiological cells and potentially affecting cytotoxicity. In this study, explored whether different concentrations of APTES silane in cationic coating on titanium samples affect electrostatic charging behavior and its subsequent effects on interactions with microbiological and mesenchymal stem cells.

## 2 Materials and Methods

### 2.1 Experimental Design

To produce the cationic coatings, titanium discs were first activated by alkalization with NaOH using the PEO technique (step 1). The samples were subsequently functionalized by immersion in 3-aminopropyltriethoxysilane (APTES) (Step 2) to enhance their positive charge. Figure 1 illustrates the experimental design of this study. Five groups were tested: untreated Ti (control), PEO (treatment control), PEO+APTES0.3 (experimental 1), PEO+APTES0.6 (experimental 2), and PEO+APTES0.9 (experimental 3). The APTES concentrations for the experimental groups were 83.8 mM (0.3 mL), 167.6 mM (0.6 mL), and 251.4 Mm (0.9 mL), respectively. In the final stage, the influence of these treatments was investigated based on physicochemical properties, microbiological interactions, and cytocompatibility.

**Figure 1:**
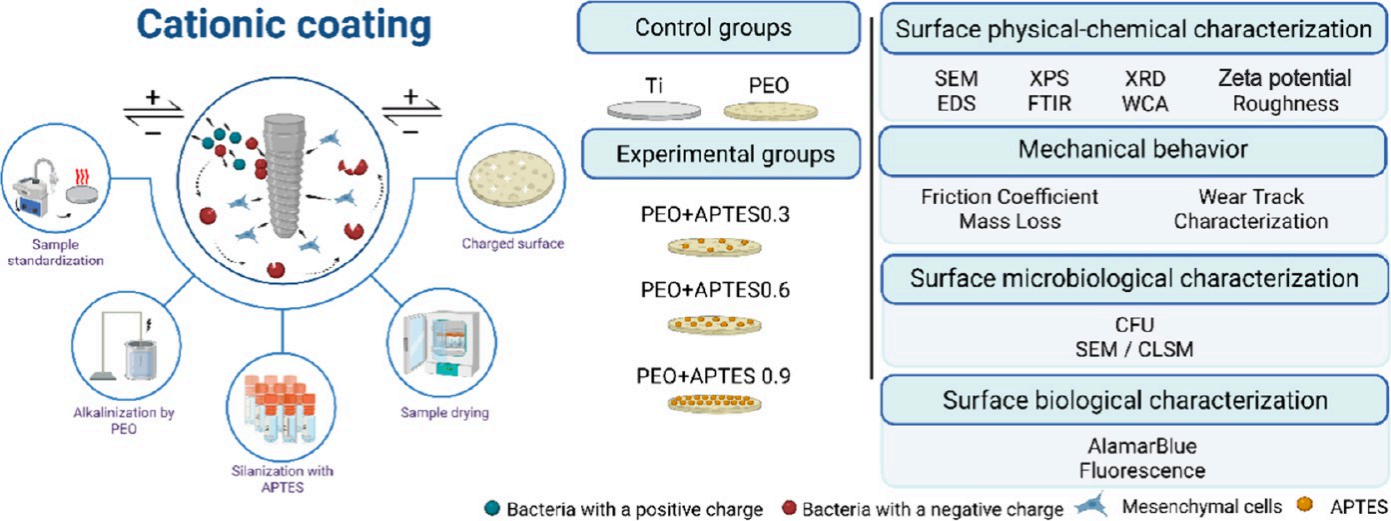
Schematic diagram of the study’s experimental design. Ti = titanium; PEO = plasma electrolytic oxidation; APTES = 3-aminopropyl-triethoxysilane; SEM = scanning electron microscopy; EDS = energy dispersive X-ray spectroscopy; XPS = X-ray photoelectron spectroscopy; FTIR = Fourier transform infrared spectroscopy; XRD = X-ray diffraction; WCA = water contact angle; CLSM = confocal laser scanning microscopy; CFU = colony-forming units. Figure created by BioRender.com (license number: FZ28N9GUYL).

### 2.2 Ti Surface Preparation

Commercially pure titanium (cpTi) discs [Grade 2, American Society for Testing Materials (ATSM)] measuring 10 mm × 1 mm (diameter-thickness) (Realum Industria e Comercio de Metais Puros e Ligas Ltd., Brazil) were used. All discs were included in self-curing resin and polished with sequential metallographic sandpaper (#320 and #400) on both sides (Carbimet 2; Buehler, USA), cleaned and degreased in a sequence of deionized water and enzymatic soap (10 min), deionized water (10 min), and 70% propyl alcohol (10 min).^12,22^ Finally, the samples were dried with jets of hot air. Polished surfaces (named Ti) were used as control for the material.

#### 2.2.1 Surface Alkalinization by Plasma Electrolytic Oxidation (PEO) (Step 1—Alkalinization)

To produce the alkalinized surfaces, the PEO technique was selected.^12^ For this, a continuous current power supply (Plasma Technology Ltd. a., China) was used. Ti discs were immersed in a tank made of stainless steel and covered in an electrolytic solution containing 0.3 M calcium acetate [Ca­(CH_3_CO_2_)_2_, Dinamica lab, Brazil], 0.02 M disodium glycerophosphate (C_3_H_7_Na_2_O_6_P, Sigma-Aldrich, USA) and 0.4 M sodium hydroxide (NaOH, Sigma-Aldrich, USA). The treatment was carried out at a voltage of 500 V, a frequency of 1000 Hz, and a duty cycle of 40% for 5 min. This process allows microdischarges generated between the sample (anode) and the solution which produce rupture of the amorphous TiO_2_ layer. As a result, the bioactive elements Ca and P, along with hydroxyl radicals (−OH), can be incorporated into the surface, allowing the formation of the crucial functional group required for silanization, which is then performed using a covalent bond. Finally, PEO-treated surfaces were washed in deionized water and dried at room temperature for 24 h.

#### 2.2.2 APTES Functionalization on the PEO-Treated Surfaces (Step 2—Silanization)

For the silanization step, the alkalinized surfaces were immersed in solutions containing different concentrations of 3-aminopropyltriethoxysilane (APTES, Sigma-Aldrich, USA) (83.8, 167.6, or 251.4 mM) and 15 mL of tetrahydrofuran (THF, Quimesp Química Ltd.a., Brazil) for 1 day at room temperature.^12,14^ This process, carried out with different concentrations, promoted positive charging on the surface of the samples. After immersion, the positively charged surfaces were washed with methanol (methyl alcohol, Labsynth, Brazil) and chloroform (chloroform, Sigma-Aldrich, USA), with three repetitions of each reagent, and incubated in vacuum for 1 h at 80 °C.^12^ Thus, the following experimental groups were obtained: PEO+APTES0.3, PEO+APTES0.6, and PEO+APTES0.9.

### 2.3 Surface Characterization

#### 2.3.1 Surface Morphology, Composition and Charge

The morphological analysis of the surfaces was conducted at magnifications of 1000× using a scanning electron microscope (SEM) (JEOL JSM-6010LA, Japan). The electron beams were used at low accelerating voltages (3 kV).^11^ Then, the samples were investigated regarding their composition to highlight the elements that may be associated with the increased load, particularly the presence of −OH (Oxygen levels) and APTES groups (Carbon and Silicon levels) on the surfaces. Therefore, energy dispersive spectroscopy (EDS; Bruker, Germany) was used to detect the presence of each chemical element. The test was carried out in order of 1 μm^3^ and displayed on a color map.^23^ Then, an X-ray photoelectron spectroscope (XPS; Vacuum Scientific Workshop, VSW HA100, Manchester, United Kingdom) was used to evaluate the chemical state of the outermost oxide layer of the samples, operating under a measuring pressure of less than 2 × 10^–8^ mbar and angle of 90° with a maximum sampling depth of 15 Å. The electrons were excited and irradiated at Al Ka, 1486.6 eV for a time of 150 s. The C1a line, with a binding energy of 284.6 eV and pass energy of 44 eV, was used to correct the charge effects.^12,23^ Furthermore, the atomic ratios of the identified elements were determined by Gaussian deconvolutions. To validate the surface molecular structures required to obtain the charged surfaces, Fourier transform infrared spectroscopy (Jasco FTIR 410 spectrometer, Japan) was used, where the spectra were averaged from 128 scans acquired with a resolution of 4 cm^–1^.^12^ The surface zeta potential was measured using a SurPASS Electrokinetic Analyzer manufactured by Anton Paar GmbH in Graz, Austria. The samples were attached to a clamping cell and placed into the SurPASS instrument. The clamping cell was adjusted to maintain a channel height of approximately 100 μm. The system was flushed with a 0.001 mol/L NaCl electrolyte solution, and the pH was set to 7.4.^24^ The streaming surface zeta potential was measured three times in the same sample to ensure accuracy.

#### 2.3.2 Crystallinity, Roughness and Wettability

An X-ray diffractometer (XRD; Panalytical X’ Pert3 Powder, UK) was used with CuKα (λ = 1.540598°Å), power of 45 kV with 20 mA current at a speed of 0.02°/s, fixed angle of 2.5° and variations between 30° and 90° to analyze the crystalline phases of the oxides formed on the surfaces by the Grazing Incidence method.^12^ Surface roughness was obtained using a profilometer (Dektak 150-d; Veeco, NY, USA), applying a cut-off point of 0.25 mm and a measurement speed of 0.05 mm/s across the right, central, and left regions of the disc for a duration of 12 s.^23^ Surface polarization was assessed by a wettability test, measuring the contact angle between a water droplet (5 μL) and the disc surface. A goniometer (Ramé-Hart 10000; Ramé-Hart Instrument Co, USA) associated with the software (DROP image Standard, Ramé-Hart Instrument Co, USA) was used for this, employing the sessile drop technique which was measured after the drop made contact with the surface.^25^

### 2.4 Surface Stability and Tribological Behavior

To evaluate the surface stability, a 28-day immersion protocol in simulated body fluid (SBF) was carried out. Each sample (*n* = 3 per group) was placed in a cryogenic tube containing 1 mL of SBF, sealed, and kept in an incubator at 37 °C for 7 days, followed by 7 days at room temperature. The cycle was repeated once, inducing temperature fluctuations. The SBF used had an ionic composition similar to human plasma, with the pH adjusted to 7.4, and it was not renewed during the immersion period. After the experiment, the samples were analyzed for morphology by SEM, chemical composition by EDS, and functional groups by FTIR, assessing changes such as delamination, variations in element distribution, and preservation of characteristic peaks. This protocol allowed verification of the structural and chemical stability of the surfaces under conditions simulating the physiological environment, ensuring reproducibility of the results.

The mechanical resistance of the surface was assessed using a customized tribological system (pin-on-disk tribometer, School of Mechanical Engineering, University of São Paulo, São Carlos, SP, Brazil), as described previously.^12,23^ A constant normal load of 1 N, a track diameter of 7.0 mm, a sliding speed of 0.01 m/s, and a test duration of 100 s were applied. Each test was conducted in 100 mL of SBF at 37 °C and pH 7.4 to mimic the composition of blood plasma. Mass loss (mg) was evaluated using a precision balance (AUY-UNIBLOC Analytical Balance, Shimadzu Corporation, Kyoto, Japan) by recording the disc mass before (baseline) and after the tribological tests. Surface morphology and wear scars were examined using SEM (JEOL JSM-6010 L A, Peabody, MA, USA). The wear area was quantified with an optical microscope (VMM-100-BT; Walter UHL, Asslar, Germany) coupled to a digital camera (KC-512NT; Kodo BR Eletrônica Ltd.a., São Paulo, SP, Brazil) and an analysis unit (QC 220-HH Quadra-Check 200; Metronics Inc., Bedford, MA, USA). The total surface area was calculated from the disc edge measurements using the formula 2π*rd* + π*d* ^2^, where π = 3.14, *r* is the inner disc radius, and d is the width of the wear track (intraclass correlation coefficient = 0.879; *p* < 0.0001).

### 2.5 Microbiological Test

#### 2.5.1 Microbial Adhesion, Biofilm Formation, and Cell Viability

To verify the electrostatic interaction in different bacterial strains, tests were carried out using two monospecies biofilm models. *Staphylococcus aureus* (*S. aureus*, ATCC 25932) was selected as the Gram-positive strain, and *Escherichia coli* (*E. coli*, BL21) as the Gram-negative strain. Both microorganisms were separately reactivated on Mueller-Hinton (MH) agar plates (Becton-Dickinson, USA), and incubated for 24 h at 37 °C in a 10% CO_2_ atmosphere.^13^ Subsequently, approximately eight colonies of each strain were collected and cultured overnight (14 h) in 5 mL of MH broth at 37 °C under the same conditions. The following day, a 1 mL aliquot of the overnight culture was transferred to 9 mL of fresh MH broth and incubated for additional 4 h, allowing the bacteria to reach the exponential phase.^12^ The inoculum was then adjusted to an optical density (OD) of 0.3 for *S. aureus* and 0.1 for *E. coli* at 550 nm, corresponding to approximately 10^7^ microbial cells/mL.

Prior to bacterial cultivation on the titanium discs, the samples were sterilized by exposure to UV light (4 W, λ = 280 nm, Osram Ltd., Germany) for 20 min on each side. The sterilized discs were then arranged in 24-well plates, where each well was seeded with 100 μL of bacterial inoculum and 900 μL of MH broth for biofilm formation.^12^ The plates were incubated under the same conditions as the bacterial reactivation (37 °C with 10% CO_2_) for 1 h to evaluate initial microbial adhesion and for 24 h for biofilm formation. Following incubation, the discs were washed twice with 0.9% NaCl to remove the nonadherent microorganisms. They were then transferred to cryogenic tubes containing 1 mL of 0.9% NaCl, kept on ice, vortexed for 15 s, and sonicated (Branson, Sonifer 50, Danbury, CT, USA) at 7 W for 30 s. A 100 μL aliquot of the sonicated bacterial cell suspension was serially diluted (7-fold) in 0.9% NaCl, plated onto MH agar, and incubated for 24 h. Colony-forming units (CFU) were subsequently counted. Additionally, the biofilm morphology was analyzed using SEM (JEOL JSM-6010LA, Japan) operating at 15 kV. For this purpose, the strains were fixed on the discs using 2.5% glutaraldehyde for 4 h, followed by dehydration in 50, 70, 90, and 100% alcohol for 10 min each. Furthermore, to indirectly assess the effectiveness of cationic coatings in disrupting bacterial membranes through strong electrostatic interactions, cell viability was analyzed using confocal laser scanning microscopy (CLSM, Olympus, FV4000, Japan). For fluorescence analyses, the samples were stained with Live/Dead BacLight Bacterial Viability Kit L7012 solution (Invitrogen-Molecular Probes, USA) using 1.5 μL/mL SYTO-9 reagent (485–498 nm; Thermo Scientific, USA) and 1.5 μL/mL propidium iodide solution (490–635 nm). Living cells were stained green, while dead cells were stained red.^13^

### 2.6 Biological Cytocompatibility

#### 2.6.1 Cell Culture

The cytocompatibility of the developed surfaces was assessed using cryopreserved rat mesenchymal stem cells (rMSC SCR027, Sigma-Aldrich, USA). The cells were cultured in α-MEM culture medium (Gibco, Life Technologies, USA) supplemented with 10% fetal bovine serum (FBS) and 1% streptomycin until reaching confluence (∼7 days) in a cell culture incubator at 37 °C with 5% CO_2_.^23^ Prior to cell seeding, the titanium discs were sterilized by UV light, placed in a 24-well plate, and seeded with 60 μL of rMSC at a concentration of approximately 1.08 × 10^2^ cells/mL. The cells were allowed to adhere for 60 min under incubation conditions. Then, 540 μL of supplemented α-MEM was added to each well, and the plates were returned to the incubator under the same conditions for further analysis.

Cell viability was evaluated using the Resazurin salt (AlamarBlue) assay after 1, 3, and 8 days of cell culture.^21^ The culture medium was removed, and a solution of Resazurin salt (Resazurin sodium salt, Sigma-Aldrich, USA) in fresh medium at a concentration of 15 μg/mL was added to the wells. The plates were incubated for 4 h, following the manufacturer’s instructions, to allow the colorimetric change of Resazurin to Resorufin. After incubation, 100 μL of the solution was transferred to a 96-well plate for absorbance measurements at 570 nm (reduction) and 600 nm (oxidation). In addition, a microscopic cell count assay using fluorescence staining was performed on days 1 and 3. Cells were stained with DAPI (4′,6-diamidino-2-phenylindol) diluted in Phosphate Buffered Solution (PBS) at 1 μL/mL.^26^ The discs were carefully washed in PBS, and the cells were fixed in methanol, followed by washing in bovine serum albumin (BSA), and PBS. The staining, the cells were treated with 1% Triton for 30 min to permeabilize the cell membranes, followed by DAPI staining for 10 min. After staining, the cells were washed in PBS and protected from light until visualization using fluorescence microscopy with a structured light camera (Nikon, TI Eclipse, Japan) and an epifluorescence microscope (Leica DM IRB, Japan). For all experiments, the culture medium was refreshed every 2 days.

### 2.7 Statistical Analysis

Data analysis was performed using IBM SPSS Statistics software (IBM SPSS Statistics for Windows, v.21.0. IBM Corp., USA). The Shapiro-Wilk method was used to verify the normality of the data, while the Levene test was applied to assess homoscedasticity for response variables. Based on the results, a one-way ANOVA flowed by the Tukey HSD test was used to verify differences across the groups for each dependent variable, considering a significance level of *p* < 0.05. Graphs were generated using GraphPad Prism 8.0.2 (version 8.0.2.263, GraphPad Software, USA).

## 3 Results and Discussion

### 3.1 Physicochemical Characteristics Are Altered after Loading Charge on Ti Surfaces

The morphology and chemical characteristics of implant surfaces are crucial for their long-term success. SEM micrographs (Figure 2a) revealed distinct morphological differences between the Ti group and the functionalized groups. The Ti group exhibited a smooth and polished surface, indicating that the initial polishing process effectively standardized the samples. In contrast, the PEO-functionalized groups displayed a porous and uniformly rough surface, a characteristic attributed to the microdischarges generated during the PEO process.^21^ This topographical modification aligns with findings from other studies on titanium discs.^20,27^ The treatment involves the application of high tension, which generates an electric current in an electrolytic solution. This electric current causes plasma microdischarges, which occur at high voltages. These discharges break up the titanium oxide (TiO_2_) layer on the surface of the material, altering its properties.^28^ This process creates high temperatures, enabling bioactive elements such as Ca, P, and OH to integrate into the surface and form a dense oxide layer.^28^ Upon completion of the electrolytic process, pores are formed. The resulting porous structure enhances surface roughness, addressing the limitation of smooth surfaces that can compromise the primary stability of implants.^29^ Additionally, this increase in roughness increases the actual surface area available to react with the silanes, favoring a more efficient and robust silanization process. The increased roughness not only improves implant stability but also mimics bone-like structures, which significantly promotes osseointegration—an essential factor in implant success.^30^ Following the functionalization process, silanization with APTES, regardless of the concentration used, did not alter the morphology formed after PEO treatment. It is important to note that the films formed by organosilanes have thicknesses on the nanometer scale.^31^ In the specific case of APTES, films formed consist of a molecular structure with 2-D quasicrystalline characteristics.^32^ This organization involves the interaction between the Si groups and the −OH groups on the surface, resulting in the formation of siloxane covalent bonds (Si–O–Si) without completely blocking the pre-existing pores.^33^ In addition, the technique used to deposit the silane provides precise control over the film formed, resulting in a charged surface without altering the initial topography^33^

**Figure 2:**
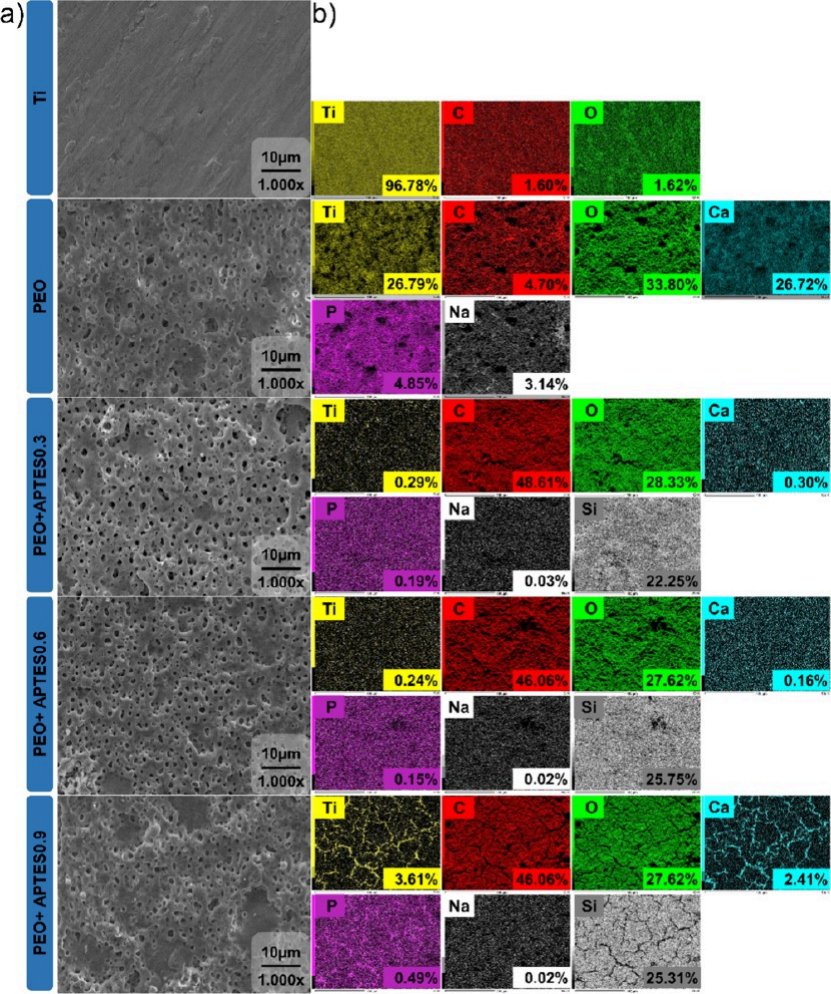
Surface morphology and chemistry. (a) Representative SEM micrographs (1000× magnification was used to visualize morphological changes) (*n* = 3/group). (b) EDS maps and individual elements with the atomic percentage of elements (*n* = 1/group).

To confirm the biofunctionalization via PEO, EDS analysis was performed to determine the chemical composition and identify elements incorporated during the electrolytic process. The colorimetric maps (Figure 2b) reveal the presence of titanium (Ti), carbon (C) and oxygen (O) across all analyzed groups. The reduction in the apparent concentration of Ti after silanization is due to the formation of a thin molecular film that covers the surface of the material, making it difficult to detect titanium directly using techniques such as EDS. These findings are in agreement with previously reported results.^12,34^ In contrast, an increase in carbon concentration is observed, attributed to the presence of abundant methylenic chains (CH_2_) in the silane used.^35,36^ As the film uniformly covered the surface, higher atomic proportions of carbon were detected, reflecting the composition of the coating applied. A noticeable increase in the oxygen concentration was observed for the groups that were anodized, which remained stable after silanization with APTES. This element has a key role for forming hydroxyl functional group (−OH), which is essential for the covalent bond between Si–O.^12^ In addition, the analysis confirmed the incorporation of bioactive elements into the oxide layer, such as Ca and P. These two ions are components of bone tissue and play a significant role in the activation of osteoblasts during osteogenesis processes.^28^ However, after treating the samples with APTES, there was a reduction in the concentration of Ca and P on the surface. This phenomenon may have occurred due to competition between the Si molecules and the Ca^2+^ and PO_4_^3^- ions for the free −OH groups on the surface.^37^ During the silanization process, the APTES molecules react with the −OH groups present on the surface, forming siloxane-type covalent bonds (Si–O–Si), which are more stable and stronger.^33,38^ As a result, the previously adsorbed ions are replaced, reducing their detectable presence on the surface of Ca and P. Additionally, the APTES film may have interfered with the detection of Ca and P, as the formed layer may have reduced the visibility of these elements in the superficial layers of the analyzed sample. Sodium (Na) was also detected due to the NaOH solution used in the PEO process. Finally, the mapping identified silicon (Si), derived from the APTES molecule, confirming successful functionalization.^12^ The presence of Si suggests that the ethoxy groups (−OEt) of APTES reacted with hydroxyl groups −OH on the surface, forming Si–O–Si bonds, which indicate covalent bonding of APTES to the alkalinized surface.^14^ Notably, the Si percentage remained similar across functionalized groups, regardless of the APTES concentration, suggesting uniformity in functionalization.^14^ It is important to note that, although different concentrations of APTES were used, the amount of the amount of OH groups remained the same for formation of the silicon based. Thus, as the detection of O was similar in all the groups, the Si–O covalent bonds were formed based on the availability of these functional.^33^ In other words, once all −OH groups were saturated, any excess Si had no available binding sites, limiting the further incorporation of Si.

The composition of the external oxide layer of the samples was investigated by means of XPS analysis, as shown in Figure 3a. The elements present were determined by electron emission, and the spectrum was previously calibrated with carbon (C 1s). It should be noted that the calibration process may introduce small variations in the eV values. The O 1s peaks observed in the PEO-treated group indicate the presence of −OH ions, as well as the formation of O–Ti–O bonds in the TiO_2_ layer, resulting from the oxidation of titanium during the treatment.^27^ In the APTES groups, peaks were identified at approximately 533 eV, suggesting the formation of Ti–O–Si bonds, indicating covalent bonds between the titanium surface and the silicon molecules.^39^ Additionally, the N peaks were used to identify the surface charge trends, with a binding energy value of approximately 398 eV.^40^ This value is usually associated with amine groups, which, due to their high p*K*_a_, induce a positive charge on the surface.^12^ The lines marked in blue or red represent the evolution of the spectra’s deconvolution process. Finally, silicon was detected around 102 eV in the APTES groups, confirming the success of surface silanization.^12,40^

**Figure 3:**
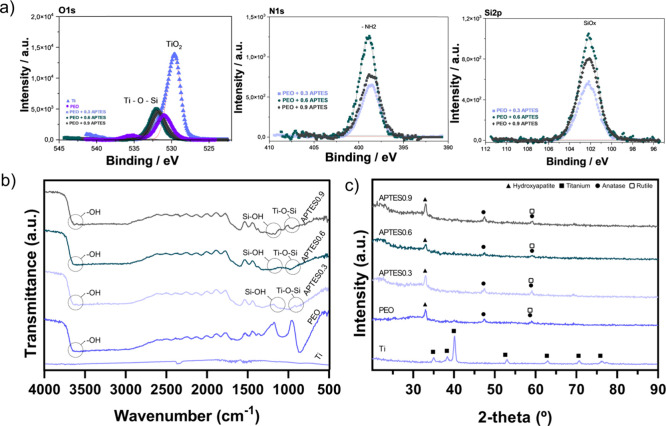
Chemical composition and microstructure of the samples. (a) Detailed XPS spectra for Ti-based control and experimental groups. The oxide binding spectra for all groups (*n* = 2/group); (b) FTIR spectra for all groups expressing the functional structure of the samples (*n* = 2/group) and (c) XRD of surfaces showing peaks of crystalline phases (*n* = 1/group).

The characterization of the chemical structure, using spectra acquired by FTIR allowed to identify the functional groups responsible for the formation of the cationic coatings. Figure 3b shows the FTIR spectra in the range of 4000–500 cm^–1^ for the different groups. The Ti group showed no bands as it was used as a baseline. Bands between 3630 and 3640 cm^–1^ were identified in all PEO-treated groups, which are associated with the bending vibration of the hydroxyl groups (Ti–OH), confirming the chemical modification of the surface necessary to promote alkalinization.^12,41^ The presence of −OH groups in the coatings is discussed in previous studies, which also reported the ability of these groups to increase surface wettability, favoring protein adsorption and consequently accelerating the cellular response.^12^ This factor is critical for successful osseointegration in biomedical implants. In addition, a higher amount of hydroxyl groups tends to increase the chemical stability of the surface, potentially prolonging the durability of the treatment.^42^ A stretch around 3400 cm^–1^ is associated with the presence of N–H bonds, indicative of amine groups on the surface.^43^ This finding suggests a tendency toward the formation of a more electropositive surface, which is of paramount importance for the development of the experimental surface in this study. In addition, amines play an important role in the stability of the treatment, due to their chemical stability and the formation of stable covalent bonds.^44^ Another relevant band identified in this study appears between 1150 and 1170 cm^–1^, indicating the presence of Si–OH bonds, which ensure the formation of siloxane bonds.^45^ The covalent bonds are fundamental, as they create a network of strong interactions between the coating and the substrate, improving coating adhesion.^46^ Molecular dynamics studies suggest that these covalent bonds, by electron sharing between the substrate and the silane, provide a more resistant barrier against corrosion in aggressive physiological environments.^12^ This has also been confirmed by previous studies.^45,47,48^ which demonstrated an increase in the durability of siloxane-based coatings in highly corrosive conditions. In addition, the appearance of peaks around 940 and 960 cm^–1^ corresponds to the Ti–O–Si covalent bond as a result of the Si (OH) condensation reaction and hydrolyzed production on the titanium surface.^45^ In some, the presence of this band confirms the functionalization of the titanium surface by APTES, indicating the formation of stable covalent bonds. Thus, the PEO process ensures precise control over the presence of functional groups on the surface, optimizing bioactivity.

The phase composition of the oxides formed on the surfaces studied was analyzed by XRD. In Figure 3c, shows that functionalization by PEO allowed groups electrolyzed in NaOH, Ca and P solution to exhibit crystalline structures in the oxide layer, revealing peaks corresponding to anatase and rutile, as well as a peak suggesting the formation of a hydroxyapatite (HAp) layer.^49,50^ It is important to note that only the most defined peaks were identified, related to the oxide matrix and the most important crystalline contributions, due to their direct influence on the surface’s functionality. These results provide valuable insights into the bioactivity of the surface, which is crucial for bone implants. The interaction of hydroxyl ions with titanium ions initiates the formation of anatase and rutile. At first, anatase is formed, which favors better interaction with the cells. The presence of anatase stimulates bone growth and, and being a more reactive structure, it improves cell adhesion.^51^ As the treatment time increases, the rutile layer forms, providing greater chemical stability and improving the surface’s mechanical properties, as well as offering greater protection against corrosive media.^52^ In addition, when OH- ions interact with calcium ions (Ca^2+^) and phosphate groups, a highly crystalline hexagonal structure is formed, allowing for easy isomorphic cationic and anionic substitutions.^53,54^ This reaction leads to the formation of HAp, the primary inorganic component of bone tissue, which increases biocompatibility and improves osseointegration.^54^ Additionally, due to its unique structure, the presence of HAp on these surfaces contributes to modulate the growth of mixed biofilms (comprising fungi and bacteria), thereby aiding in the control of peri-implant infections.^55^ Thus, the oxide formation enables the development of a surface treatment with enhanced bioactivity, which is essential for biomedical implants application.

Although XPS suggests an increase in charge due to the presence of protonated amines, surface zeta potential measurements were conducted on the samples for further confirmation. Significant variations in surface charge were observed on the different treatments applied. For the Ti group (Figure 4a), the zeta potential was −28.60 ± 0.46 mV, indicating a strongly negative surface, which is typical due to the presence of oxide layer on bare Ti material.^56^ After PEO treatment, there was a significant decrease in the surface charge near the isoelectric point, which measured −0.76 ± 0.36 mV. At the isoelectric point, there is a net balance between positive and negative charges. The reduction in zeta potential value may be due to the formation of calcium phosphate on the titanium surfaces, which competes with hydroxyl groups available for further reaction with APTES.^57^ In contrast, the addition of APTES led to a significant increase in the positive charge of the surfaces. With the higher concentration of APTES, the surface charge was 7.09 ± 1.43 mV, and this charge remained positive even at lower APTES concentrations, with values of 9.33 ± 0.50 and 7.91 ± 1.90 mV, respectively. The values indicate that the treatments produced positively charged surfaces, attributed to the appearance of protonated amine groups (−NH_3_^+^) that were identified in the measurements with p*K*_a_ below 9, corroborating Tamba et al. study.^58^ It is also worth noting that the APTES immobilization method, after PEO alkalinization, allowed further enhancement of the load increase. A previous study, which used the conventional hydrothermal method to immobilize APTES, showed lower loading results than ours, which demonstrates that the protocol for increasing the surface charge was effective, altering the ionic character of the surface.^59^ In addition to potentially increasing the antimicrobial effect, it may also improve cell adhesion. The presence of protonated amines in the cationic coating facilitates electrostatic interaction, providing the treatment with greater durability and a surface that is more resistant to adverse physiological conditions.

**Figure 4:**
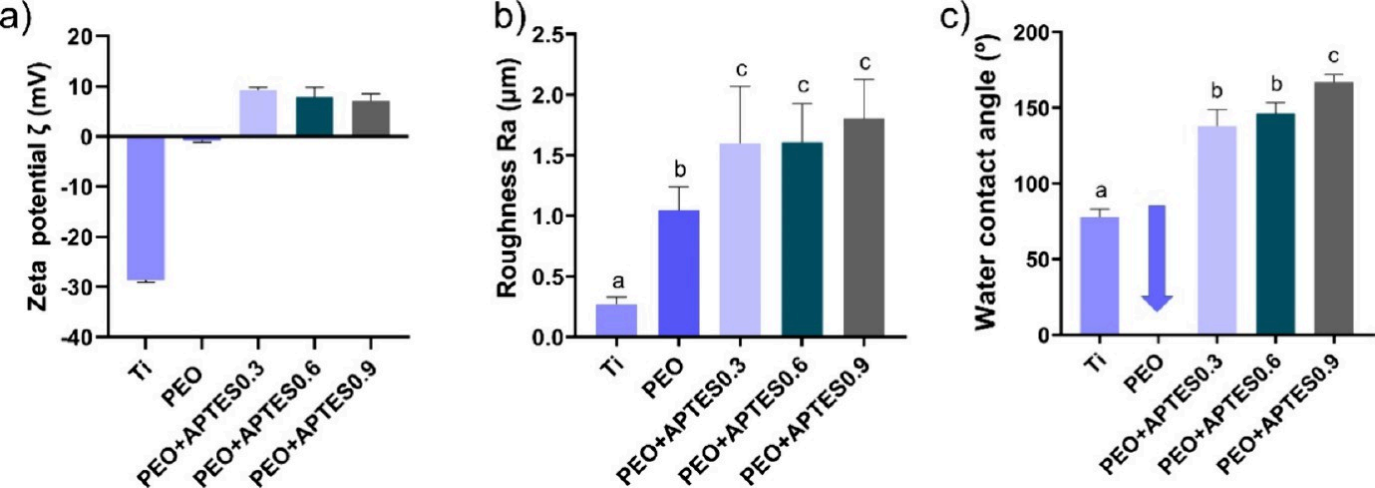
Physicochemical characterization of the control and experimental groups. (a) Zeta potential (*n* = 1/group); (b) surface roughness parameters [*R*_a_ = arithmetic roughness] by profilometry (*n* = 5/group) and (c) water contact angle (*n* = 6/group). Different letters indicate significant differences among the groups (*p* < 0.05, Tukey’s HSD test). The error bars indicate standard deviations.

When using PEO treatment to enhance the alkalization process, a porous and bioactive layer was formed on the titanium surface, resulting in increased roughness values in the groups treated by this method (Figure 4b). Additionally, silanization provided further increase in roughness values (*p* < 0.05). It is worth noting that treatment with APTES forms a nanostructural film by means of molecular bonds.^32^ However, these reactions can promote vertical polymerization on the surface of the film, resulting in increased roughness.^33^ Previous studies indicate that the solutions used in the silanization process promote the agglomeration of APTES molecules, which concentrate on the treated surfaces.^33^ These agglomerates are found on silanized surfaces with temperature protocols below 60 °C.^60^ The arithmetic roughness value (*R*_a_) was 0.28 μm for the Ti group, 1.05 μm for the PEO group, and 1.60, 1.61, and 1.81 μm for the PEO+APTES0.3, PEO+APTES0.6, and PEO+APTES0.9 groups, respectively. According to Albrektsson et al., who classified implant topography, roughness can be classified as smooth (*R*_a_ ≤ 0.4 μm), minimally rough (*R*_a_ > 0.4 - ≤ 1.0 μm), moderately rough (*R*_a_ > 1.0 - ≤ 2.0 μm) and highly rough (*R*_a_ > 2.0 μm).^61^ Therefore, the surfaces developed in this study have moderate roughness, comparable to the roughness levels of commercial implants, such as Straumann and Nobel Biocare.^61,62^ Despite the controversy surrounding rough surfaces, they can favor protein adhesion and, consequently, enhance pro-osteogenic cell adhesion, as these cells tend to favor rougher surfaces.^63,64^ Increased roughness is achieved via the appearance of higher peaks and deeper valleys, which increases the surface area and provides greater contact between the bone and the implant, aiding the osseointegration process and creating greater initial stability.^65^ In addition, both the roughness and the chemical composition of the treatment are factors that influence the wettability of the surfaces.^33,66^ Both factors influence the surfaces’ polarization, turning them hydrophilic or hydrophobic.

The surface of the Ti group showed a hydrophilic pattern, with a contact angle of 77.6° (Figure 4c). However, this polarization shifted to superhydrophilic after treatment with PEO and NaOH, making it impossible to measure the contact angle. Hydrophilic surfaces are known to improve biocompatibility, providing better cell adhesion, and facilitating integration with biological tissues.^67^ Studies in animal models indicate that these surfaces can lead to increased bone apposition, which is beneficial for successful treatments.^68^ However, more hydrophilic surfaces can also be more susceptible to bacterial adhesion, potentially increasing the risk of infections.^69^ Although hydrophilic surfaces are generally favored in the literature, the groups treated with silanization showed a shift in polarization toward hydrophobic and superhydrophobic states, typical of the behavior of organosilane films.^70^ It is worth noting that the angles were measured 2 days after the samples were obtained and stored in Petri plates (MPL, Brazil) containing silica gel pearls (Sigma-Aldrich, USA) to ensure that the surfaces were completely dry, i.e., without any water residue that could interfere with the measurements. As a result, the PEO+APTES0.3 and PEO+APTES0.6 groups showed contact angles of 138° and 146°, respectively, while the PEO+APTES0.9 group displayed the largest angle of 167°. This modification to greater hydrophobic polarization is observed by the presence of the alkyl chain in the silane’s functional group. In simple terms, this chain inhibits hydrogen bonds that would bind with hydroxyl groups, enabling the formation of an apolar interface and protecting the surface from interaction with water.^71^ In this sense, the hypothesis is that higher concentrations of silane favor a greater presence of nonmolecularly bound alkyl groups, consequently forming a more intense apolar barrier, proportional to the concentration. This demonstrated that increasing the concentration of APTES results in larger contact angles, indicating a tendency for hydrophobic and superhydrophobic surfaces, which associated with pot PEO treatment may be more effective against corrosive processes and degradation processes in aqueous medium.^12^ These surfaces help prevent titanium degradation and reduce the risk of intensifying the inflammatory response.^15^ In addition, recent studies indicate that, despite high contact angles, the cellular responses are not negatively impacted, promoting cell proliferation both in vitro and in vivo, without interfering with osseointegration.^72^ In general, hydrophobic surfaces are also capable of facilitating the adsorption of proteins due to the ease with which they displace water and the strong interactions between amino acids and the surface.^73^ Another advantage of developing surfaces with high contact angles is their ability to reduce adhesion and the formation of biofilms, acting as antifouling surfaces that help control peri-implant pathologies.^72^ Additionally, the presence of amino groups in APTES enhances the biological response, supporting improved cell adhesion.^15^

### 3.2 Cationic Coatings Enhance Chemical Stability and Mechanical Resistance of Titanium Surfaces

One of the main challenges in the development of bioactive surfaces is ensuring their chemical and structural stability over time. To assess this stability, samples from the control and experimental groups were immersed in SBF for 28 days. SEM micrographs (Figure 5a) showed the preservation of the characteristic coating morphology, with no significant signs of delamination or surface degradation. EDS analysis (Figure 5b) revealed the retention of the characteristic elements of each surface: Ti predominated in the Ti groups, while Na, P, and Ca exhibited relatively similar concentrations in the PEO-treated groups. Functionalization with APTES showed continuous presence of Si on the cationic surfaces, indicating that the organosilane network remained stable after prolonged exposure to the physiological-like medium. Additionally, FTIR (Figure 5c) spectra displayed characteristic peaks at 1060 cm^–1^ (Si–O–Si) and 850–860 cm^–1^ (Ti–O–Si), suggesting preservation of the functional groups of the chemical modification.^12,74^ Although FTIR is a qualitative technique and cannot quantify the exact extent of silane integrity, the detection of these peaks reinforces the durability of the surface modification under simulated physiological conditions. Altogether, these findings demonstrate that the cationic coating possesses essential qualities for its fabrication, including structural and functional stability, which supports its application on biomedical implant surfaces.

**Figure 5:**
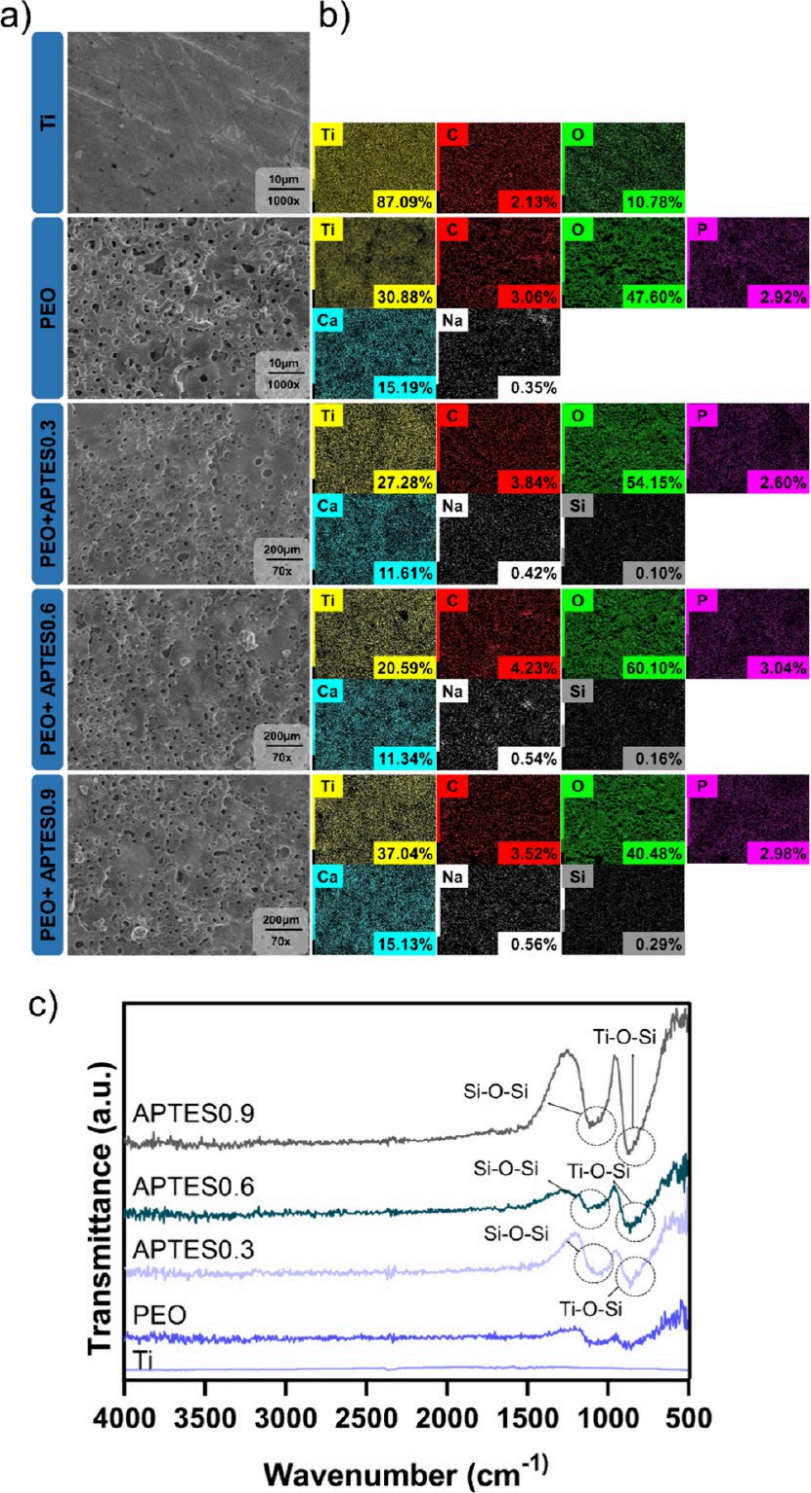
Surface stability assessment after 28 days of immersion in SBF. (a) Representative SEM micrographs (1000× magnification) showing morphological features (*n* = 2/group); (b) EDS maps and atomic percentages of individual elements (*n* = 1/group); (c) FTIR spectra of all groups showing the functional structure of the samples (*n* = 2/group).

After tribological testing, SEM images (Figure 6a) revealed that the uncoated Ti group exhibited the largest wear area (6.4 cm^2^), with evident surface degradation. In contrast, all coated groups showed reduced wear, with a progressive decrease in scar size from PEO alone (3.3 cm^2^) to PEO + APTES0.9 (1.9 cm^2^). Notably, wear in the coated groups was characterized by surface compaction rather than material removal, indicating improved abrasion resistance and mechanical durability.^21^ The reduction in wear observed for the PEO group is likely associated with the formation of a rutile TiO_2_ phase during anodization, which enhances surface hardness and contributes to greater wear resistance.^22^ Complementary to the morphological findings, EDS mapping (Figure 6b) provided further evidence of compositional stability postwear. The PEO group retained appreciable levels of calcium and phosphorus, suggesting that the anodic oxide layer remained chemically intact despite mechanical stress. Similarly, the PEO + APTES groups displayed detectable silicon content, confirming the persistence of the organosilane layer under tribological conditions. These findings underscore the chemical robustness of inorganic coatings. The improved performance of the PEO + APTES groups may be attributed to the formation of interfacial Si–O–Ti bonds, which enhance cross-linking density, increase surface stiffness, and improve structural cohesion.^12,75^ Regarding frictional behavior, the APTES-treated groups exhibited more stable friction profiles (Figure 6c) and lower average friction coefficients (Figure 6d) compared to both the Ti and PEO groups. This effect is likely due to the formation of a smoother and chemically homogeneous surface provided by the silane layer, which reduces mechanical interlocking and supports the hypothesis that the organosilane network imparts a lubricating effect while minimizing abrasive interactions.^76^ Additionally, fluctuations in the friction coefficient across all groups may be attributed to the accumulation of wear debris at the sliding interface, promoting the formation of a third-body layer that disrupts contact conditions and contributes to progressive changes in frictional response.^23^ Mass loss data (Figure 6e) followed a similar trend. The Ti group showed the greatest material loss, consistent with severe surface degradation and lack of protective coating. In contrast, treated groups demonstrated progressively lower losses, with the PEO and especially the PEO + APTES groups showing the most effective wear mitigation. This enhanced behavior is attributed to both the mechanical reinforcement provided by the Si–O–Ti network and the structural stability conferred by the hybrid architecture.^12^ Taken together, these results demonstrate that the combination of PEO and APTES treatments significantly improves wear resistance and minimizes material loss, offering durable mechanical protection under simulated physiological conditions. These findings highlight that APTES functionalization not only improves surface properties but also contributes to the mechanical performance of the coating.

**Figure 6:**
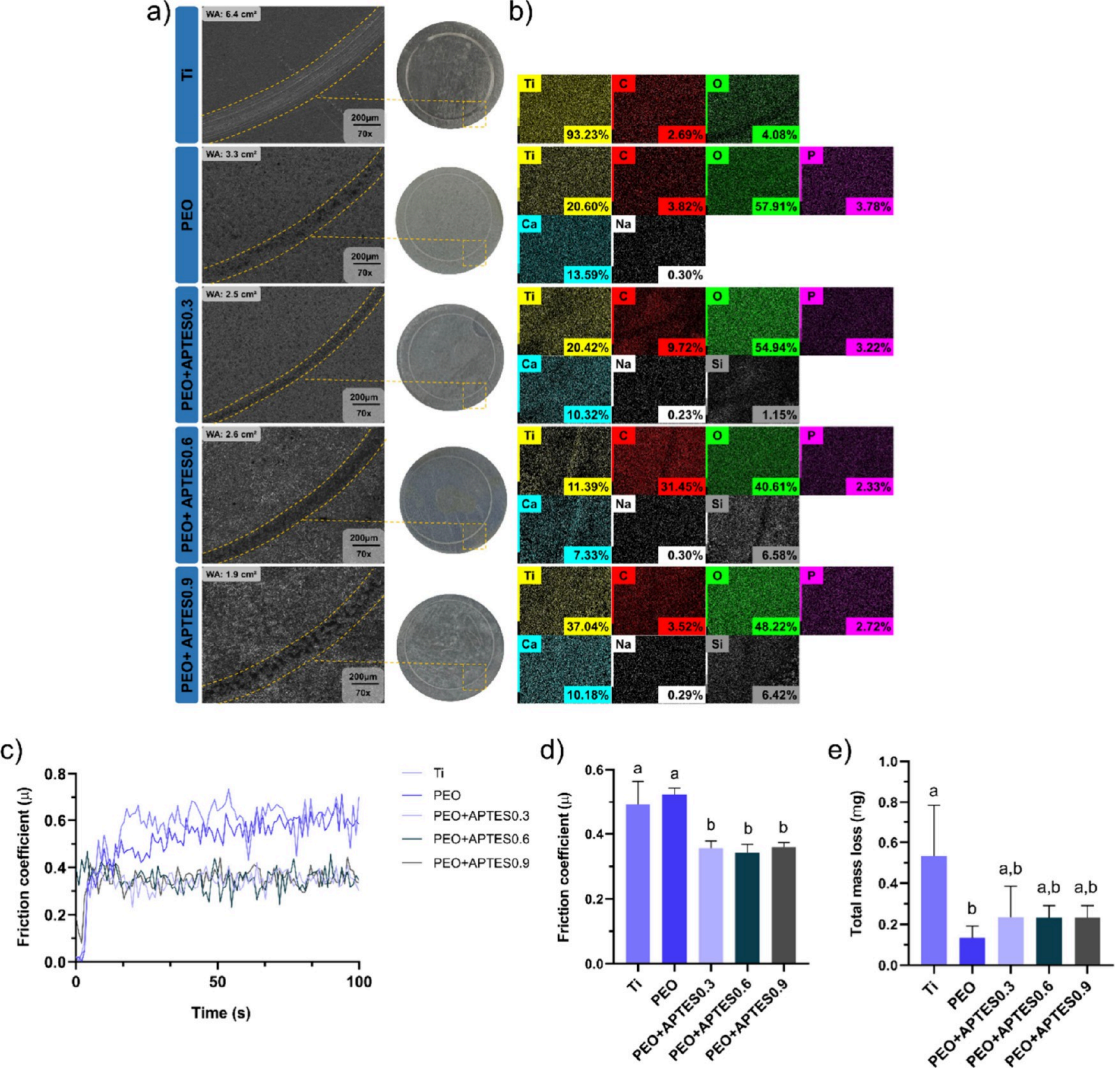
Tribological properties of the control and experimental groups (*n* = 4/group); (a) disc surfaces (right) and SEM micrographs (left) at 70× magnification showing the wear track area (dashed yellow line) (WA = wear area in cm^2^); (b) EDS color maps showing the distribution and preservation of the coating and its constituent elements, even after mechanical stress; (c) evolution of the friction coefficient for all groups during the tribological test; (d) average friction coefficient during sliding (μ); and (e) total mass loss (mg) of the samples after tribological wear. Different letters indicate statistically significant differences among groups (*p* < 0.05, Tukey’s HSD test). Error bars represent standard deviations.

### 3.3 The Electrostatic Interactions of the Cationic Coating Reduce Bacterial Adhesion and Control Biofilm Growth

Considering that biofilm accumulation can interfere with the healing process and, in advanced stages, exacerbate the inflammatory response, researchers have sought to mitigate these failures by surface treatments.^77^ Although antibiotic treatments are effective, the potential immune-resistance remains not fully understood, posing risks to the population.^78^ In this context, it was explored an effective alternative against biofilm that does not induce bacterial resistance, employing a mechanism of action based on electrostatic interaction. Microbiological tests confirmed that the immobilization of APTES on the PEO-treated titanium surface provides an antimicrobial effect. The surfaces were evaluated for initial bacterial adhesion (1 h) and biofilm formation (24 h) using Gram-positive (*S. aureus*) and Gram-negative (*E. coli*) bacterial strains in a monospecies model. It is important to note that these bacterial strains are commonly associated with more severe stages of peri-implant infection.^79^ Both bacteria have a negatively charged membranes, and share structural similarities, including a peptidoglycan layer in the cell membrane.^80^ However, Gram-negative bacteria also possess a periplasmic space and an outer membrane, which enhances their resistance to the external environment.^80^ Here, a reduction in CFU counts was observed in the positively charged APTES-treated groups compared to the Ti and PEO-treated control groups during both adhesion and biofilm formation phases (*p* < 0.0001) (Figure 7). In addition, representative micrographs of the microbial reduction observed on the treated surfaces can be visualized (Figure 7). It is hypothesized that during the initial adhesion phase, bacteria are less organized, yet more physiologically active and more susceptible to damage, which may have contributed to the antimicrobial effect.

**Figure 7:**
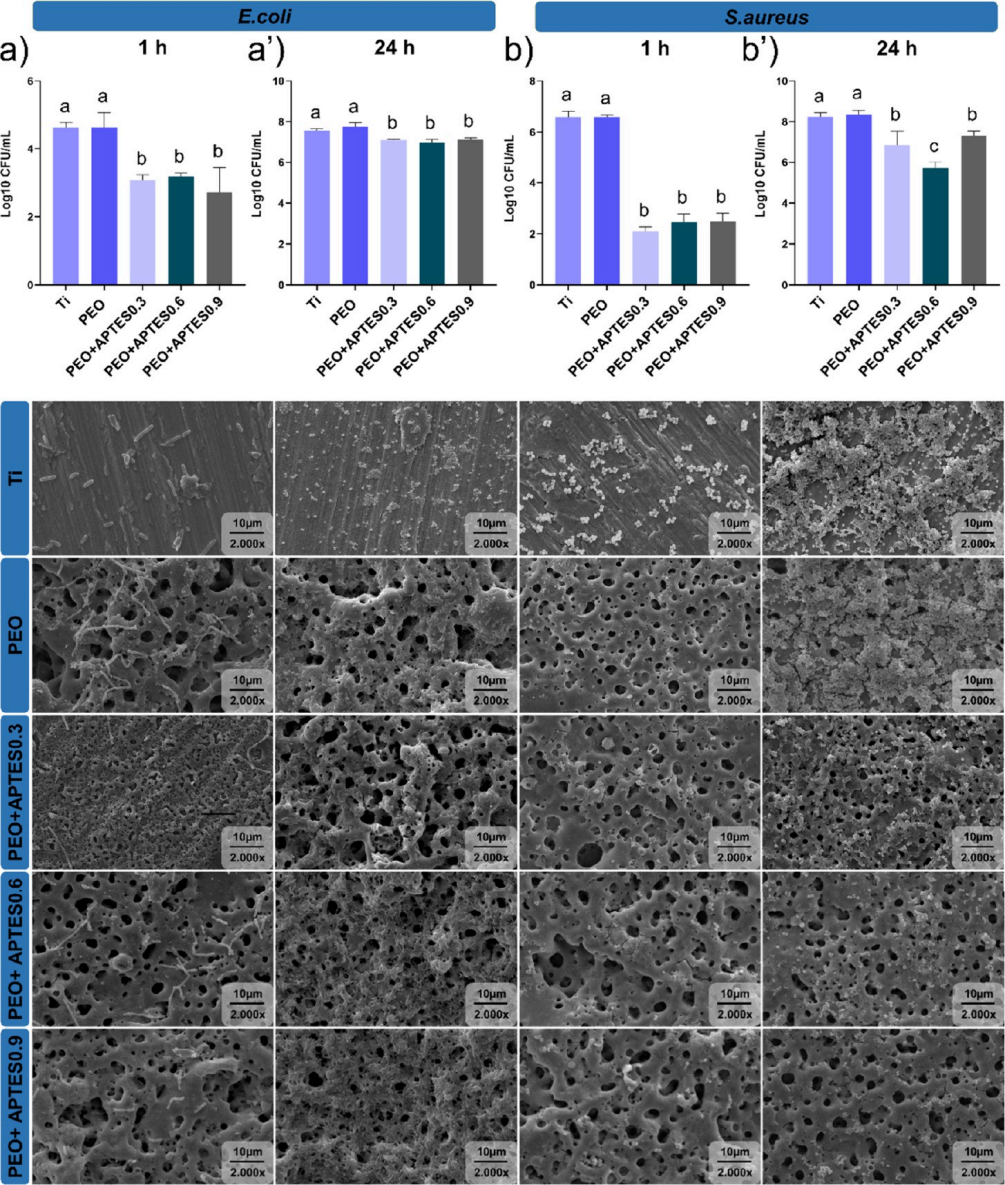
Microbiological data for control and experimental surfaces. CFU (log10 CFU/mL) (*n* = 6) and SEM micrographs illustrating bacterial adhesion and biofilm formation at 2000× magnification (scale bar = 10 μm, 15 kV) (*n* = 1): (a) 1 h adhesion of *E. coli*, (a′) 24 h biofilm of *E. coli*, (b) 1 h adhesion of *S. aureus*, and (b′) 24 h biofilm of *S. aureus*.

It should be mentioned that during the adhesion stage, a reduction of approximately 35% and 65% was observed for *E. coli* and *S. aureus*, respectively, comparing the experimental vs control groups, as can be seen in the CFU data and micrographs in Figure 7a,b. As previously mentioned, Gram-negative bacteria have greater structural resistance, which justifies the difference in percentage between the strains. Additionally, the use of silane, with its protonated and amphiphilic macromolecules containing amides and esters, modulates the adhesion and biofilm formation, being influenced by the length of the alkyl chain present in its composition, which also contributes to the prevention of bacterial adhesion.^70^ The antimicrobial effect can also be attributed to the presence of amine groups, which impart positive polarization to the surface, as confirmed by the XPS and zeta potential results.^12^ In simple terms, electrostatic interactions occur between the protonated amine (N–H) groups on the Ti surface and the negatively charged bacterial membrane, effectively inhibiting bacterial adhesion. When the bacterial membrane is negatively charged, it is strongly attracted to the surface, which can lead to membrane rupture.^14^ This disruption compromises the cell’s integrity, resulting in the release of cytoplasmic contents, and, ultimately, cell lysis.^81^ Conversely, if the membrane is positively charged, repulsion occurs.^14^ This physical principle provides a stable and long-lasting mechanism of action. The results indicate that the electrostatic interaction between cationic surfaces and bacteria significantly impacts the prevention of peri-implant infections. Even though the effect of electrical repulsion was present, we believe that the hydrophobicity of the surface contributed to these results. Studies indicate that surfaces with high contact angles perform very well during the initial adhesion phase.^72,80^ However, over time, the hydrophobic surface, which is in constant contact with ions and acids from the biofilm, may begin to reduce its hydrophobicity. In this way, we believe that hydrophobic surfaces perform better during the initial biofilm stage, where they make it difficult for bacteria to adhere, hindering their colonization. However, it is worth noting that, even when testing different concentrations of APTES, the protonation reaction is limited by the availability of molecular elements that enable siloxane bonds.^33^ Therefore, as they have similar cationic charges, as observed in the zeta potential, the antimicrobial mechanism was also similar across de APTES groups. Thus, the hypothesis is due to the fact that the layer of cations responsible for bacterial attraction or repulsion was similar between the groups, resulting in close values for both adhesion and biofilm formation.

Following the adhesion period, statistically significant differences were also observed, with an error of less than 0.01%, for biofilm formation at 24 h comparing the experimental vs control groups (Figure 7a′,b′).

To investigate whether the antimicrobial mechanism was associated with bacterial membrane disruption, a cell viability assay was performed using the Live/Dead kit. This method is based on the differential penetration of two dyes: SYTO 9, which stains cells with intact membranes (viable), and propidium iodide, which penetrates only damaged membranes, staining nonviable cells. After 1 h of adhesion, all APTES-treated groups (0.3, 0.6, and 0.9) exhibited a higher number of dead bacteria compared to the control, for both *E. coli* and *S. aureus* strains (Figures 8 and 9), demonstrating the strong electrostatic interaction between the protonated amino groups (+NH_3_^+^) and the anionic components of the bacterial membrane, such as lipopolysaccharides and teichoic acids.^15,80^

**Figure 8:**
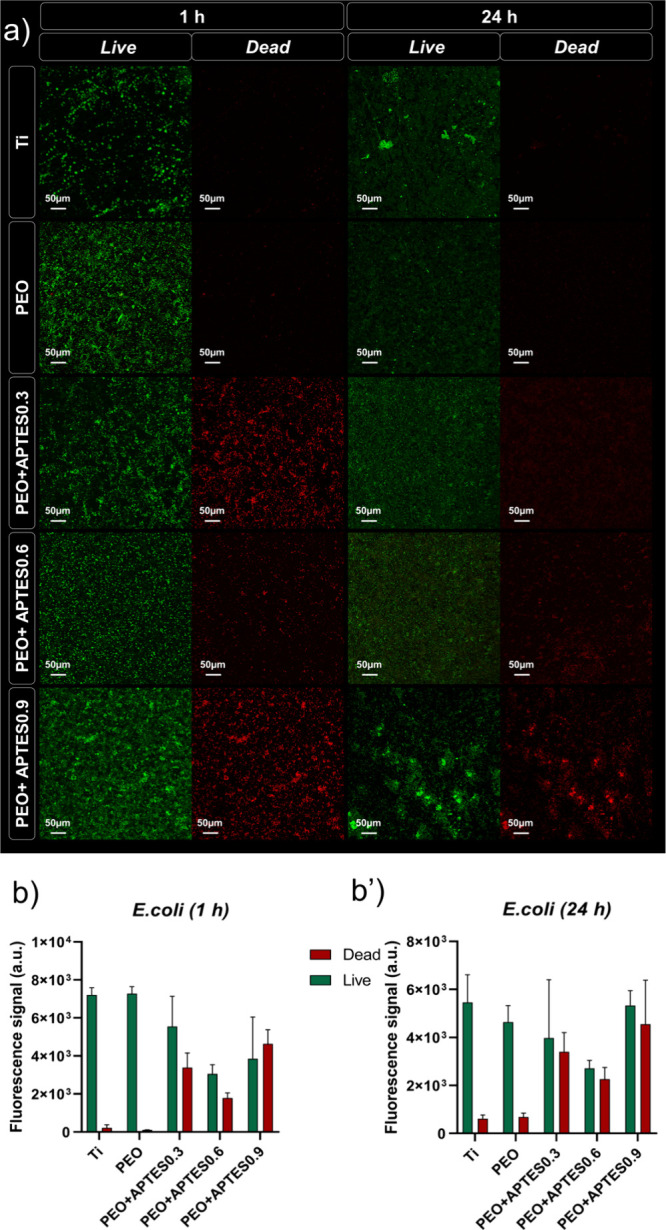
Microbiological data from the Live/Dead assay (*n* = 1/group): (a) Representative CLSM images showing the distribution of live (green) and dead (red) *E. coli* on control and experimental surfaces after 1 h of adhesion and 24 h of biofilm formation (×20, scale bar = 50 μm); (b,b′) quantification of fluorescence intensity of live and dead *E. coli* cells after 1 and 24 h, obtained from the CLSM images. Cells were quantified by counting the pixels in each image above the threshold level.

**Figure 9:**
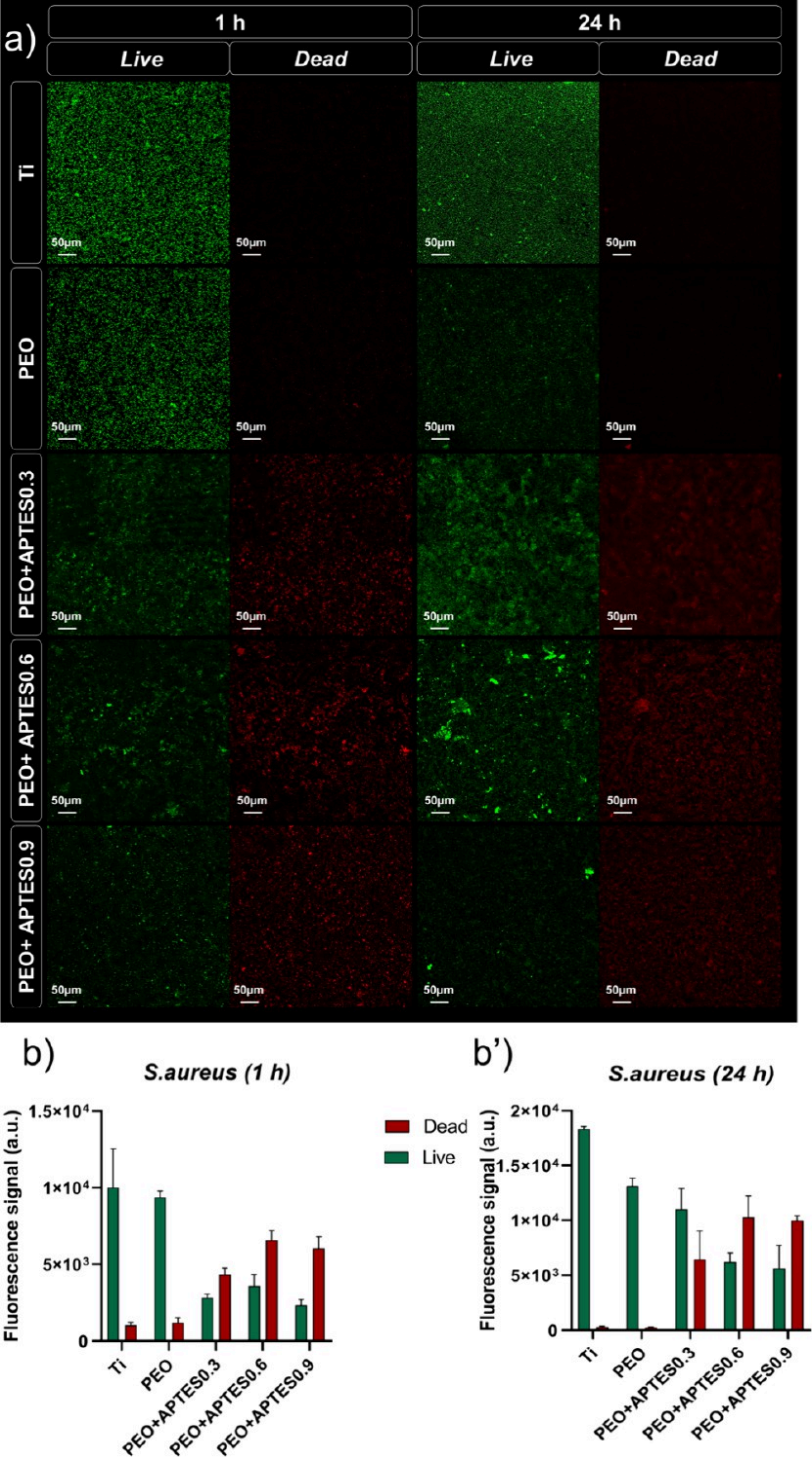
Microbiological data from the Live/Dead assay for *S. aureus* (*n* = 1/group): (a) Representative CLSM images showing the distribution of live (green) and dead (red) cells on control and experimental surfaces after 1 h of adhesion and 24 h of biofilm formation (×20, scale bar = 50 μm); (b,b′) fluorescence intensity quantification of live and dead *S. aureus* cells at 1 and 24 h, measured from the CLSM images. Cell counts were obtained by measuring the number of pixels above the threshold in each image.

After 24 h, a reduction in bacterial viability was still observed, reflecting the results obtained in the CFU assay and confirming that the antimicrobial effect persists during biofilm formation, probably due to the maintenance of the positive charge and its continuous impact on membrane integrity. However, the cationic coatings showed a higher total number of adhered bacteria compared to the control groups, as evidenced in Figure 8b,b′ for *E. coli* and Figure 9b,b′ for *S. aureus*. This finding is hypothesized to be associated with the strong electrostatic interaction between the protonated amino groups (+NH_3_^+^) on the surface and the anionic components of the bacterial membrane, favoring the capture of a greater number of bacteria present in the in vitro culture medium. In contrast, this same intense electrostatic attraction was able to compromise cell membrane integrity through direct contact, resulting in a higher proportion of nonviable cells in the positively charged groups. In addition, factors related to chemical composition, morphology, and topography may also significantly influence bacterial adhesion, modulating or enhancing these effects.^77^

Considering that the main implant failures are associated with this microbiological disorder, the reduction in biofilm observed during these periods suggests a role in preventing pathologies and controlling disease progression. It is noteworthy that the highest bacterial death occurred during the adhesion phase in both assays, when dead bacteria accumulate on the surface and consequently prevent new bacteria from coming into direct contact with the loaded surface.^82^ As a result, the effectiveness of the cationic coating begins to be reduced, since direct contact between the bacteria and the surface is necessary for electrostatic attraction or repulsion to occur.^82^ In addition, these adhered dead bacteria can contribute to the dispersion of the biofilm, as well as serving as anchoring sites and sources of nutrients for the adhesion of new bacteria.^82^

### 3.4 Protonated Amines Enhance Cytocompatibility on Titanium Surfaces

To investigate the cytocompatibility of the developed surfaces, rMSC cells were analyzed using the AlamarBlue assay (metabolic activity), a redox indicator that is reduced by metabolically active cells, resulting in a measurable color change directly correlated with cellular activity. Cell viability was also assessed using fluorescence. The AlamarBlue results on the first day revealed that the APTES groups exhibited higher metabolic activity values compared to the Ti group and similar values to the PEO-treated group (Figure 10a). On the third day, metabolic activity showed higher values (0.02 ± 0.0006) in the groups treated with APTES, demonstrating the efficacy of the proposed treatment (Figure 10a′). It is important to emphasize that mesenchymal cells are multipotent, capable of differentiating into other cell types, including osteoblasts.^83^ Osteoblastic cells, which typically possess a negative charge, can attach electrostatically to positively charged surfaces in an organized manner.^84,85^ This attachment facilitates the formation of the extracellular matrix, producing essential components for bone formation and creating a healthy peri-implant region.^86^ However, additional experiments focusing on cellular differentiation are needed to explore this topic more thoroughly. On the eighth day, statistically significant differences were observed only in the PEO+APTES0.3 group, which showed higher values (0.037 ± 0.0002), outperforming the groups with higher APTES concentrations (Figure 10a″). Conversely, the PEO+APTES0.6 and PEO+APTES0.9 groups showed a decline in cell proliferation potential, with values lower than those of the control groups (Figure 10a″). Although the APTES hydrolysis process occurs quickly, the presence of water can regulate this reaction.^87^ In this sense, based on the results obtained in this study and the evidence available in the literature, we hypothesize that higher concentrations of APTES may undergo hydrolysis over time, producing byproducts such as ethanol and silanol groups. These byproducts are released slowly, potentially reducing cell metabolism over time. Moreover, cellular responses to chemical stress of this nature are not immediate, and the accumulation of damage over time can ultimately result in cell death.^88^ Moreover, it can be hypothesized that subsurface regions may contain higher amounts of silane, acting as reservoirs of hydrolysis byproducts such as silanol groups and organic residues. Although the silane concentrations initially appear to be similar among the samples, as detected by EDS, it is important to emphasize that this technique provides averaged information from depths of approximately 1–2 μm, thus reflecting the overall surface composition but without distinguishing the outermost layers from the inner ones.^89^ In this context, the excess silane present could induce local chemical stress, compromising plasma membrane integrity and reducing cellular metabolic activity. Additionally, denser and structurally disorganized silane layers may hinder proper cell adhesion, creating microenvironments unfavorable to proliferation.^15^ These effects, combined with the potential impact of residual ethanol, offer a plausible explanation for the higher proportion of nonviable cells observed in the groups with greater amounts of silane.

**Figure 10:**
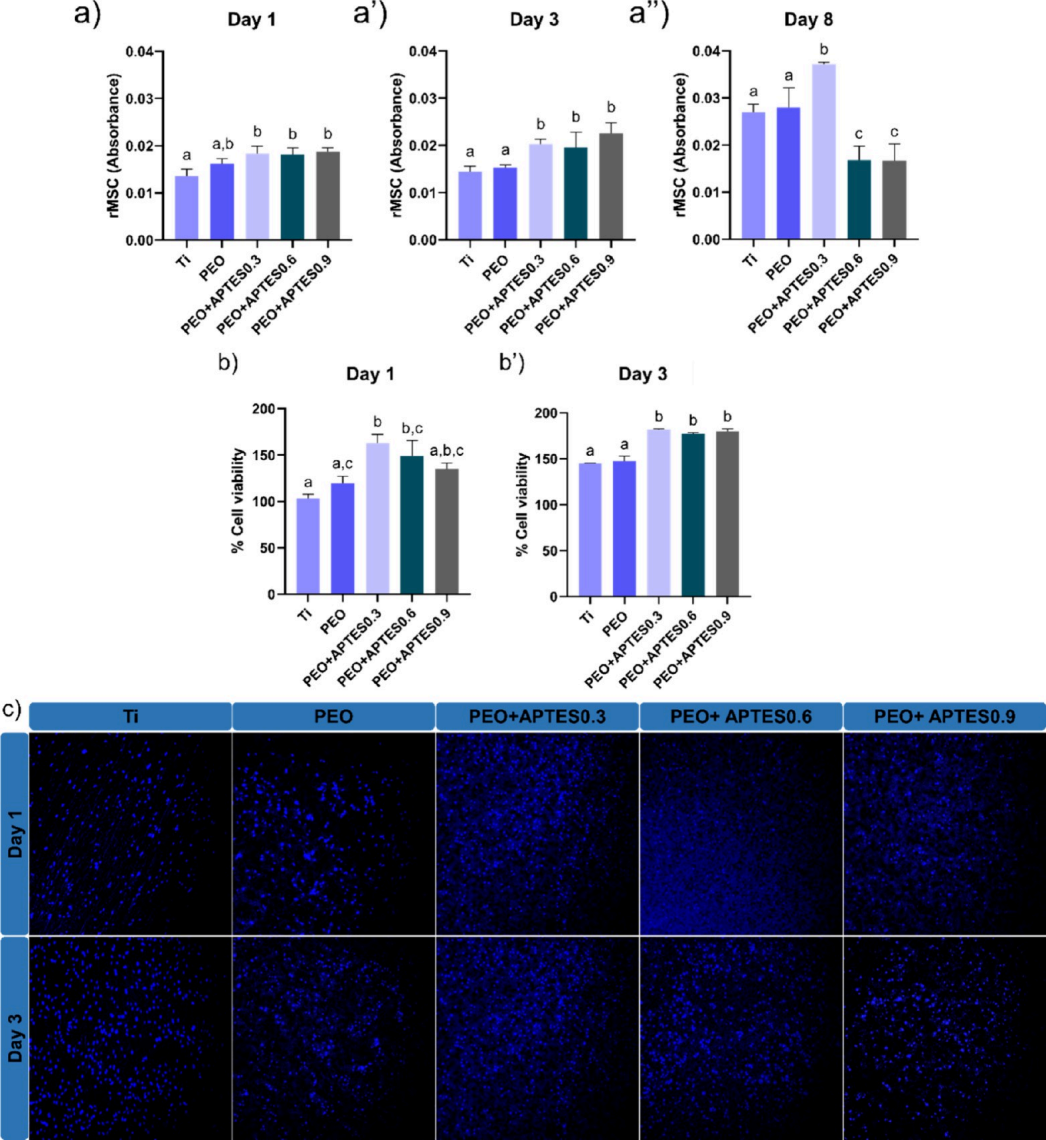
Cell viability assay. (a) Cell proliferation assessed by metabolic activity at 1 day, (a′) at 3 days and (a″) at 8 days; (b) cell count by fluorescence at 1 day, (b′) at 3 days by % cell viability and (c) cell viability by fluorescence at 1 day and 3 days. Different letters indicate significant differences among the groups (*p* < 0.05, Tukey’s HSD test). The error bars indicate standard deviations.

Consequently, when cell viability was assessed using fluorescence on the first day, the PEO+APTES0.3 experimental group stood out compared to the control groups, while the PEO+APTES0.6 and PEO+APTES0.9 groups showed comparable results, as illustrated in Figure 10b (percentage of cell viability). On the third day, cell viability was similar between the experimental groups, differing statistically from the controls (*p* = 0.0100) in Figure 10b′,c (microscopic image). For both cellular evaluation methods, the increased performance—already mentioned and shown in the respective figures—is attributed to the presence of amine groups, which directly promote cell adhesion, proliferation, and differentiation.^90^ In addition, NH_2_ groups may enhance the biological response by facilitating binding to cell receptors, thus influencing cell adhesion and the formation of functional cell structures.^15^ For the PEO group, the presence of Ca and P may have contributed to the observed results, as both elements are known to support cellular response.^28^ Furthermore, the superhydrophilicity of the PEO-treated surface likely facilitated cell adhesion by promoting interactions with phospholipids.^91^ It is worth noting that the PEO treatment increased roughness, increasing the surface area and creating an optimal environment for cell growth.^12^

In summary, the application of lower concentration of APTES proved to be the most effective, demonstrating an approximate 80% increase in cell metabolism over 8 days. The sustained high cell viability indicates that the cationic coating not only supports the initial cell adhesion but also promotes their proliferation over time. This cellular behavior is particularly relevant in implant rehabilitation, as robust cell proliferation without signs of cytotoxicity reflects excellent compatibility of the material with the oral environment. This minimizes the risk of implant rejection and postsurgical complications.

### 3.5 Establishing Cationic Coatings as a Preventive Alternative for Peri-Implant Diseases: Challenges and Perspectives in Their Production

Our findings indicate that cationic coating using APTES hold great potential for controlling peri-implant pathologies. However, since this study was conducted in vitro, it did not assess microbiological interactions with the surface in a dynamic biological environment, as would be the case in in vivo models. Such models would introduce additional complexities, including interactions with body fluids, proteins, and the immune response, which were beyond the scope of this research. Additionally, the tests with cells and bacteria were performed in isolation, without coculture, which limits the ability to fully assess the multifunctional properties of the treatment. While coculture models could provide a more comprehensive understanding of the interaction between cells and bacteria, they were not included in this study. Moreover, direct analyses using gas chromatography coupled with mass spectrometry (GC-MS), as well as indirect analyses by Fourier-transform infrared spectroscopy (FTIR), did not exhibit sufficient sensitivity to detect the byproducts generated during the silane reaction, such as ethanol and silanol groups, likely due to the low concentrations involved and the technical limitations associated with this specific system. Nevertheless, such analyses remain crucial for a deeper understanding of the mechanisms governing APTES interaction with the surface and its subsequent cellular effects. We acknowledge this limitation and plan to develop more sensitive analytical methodologies in future studies to further explore the proposed hypotheses.

Although the results obtained provide strong evidence of the treatment’s stability and durability, these characteristics have not been assessed over a long-term, necessitating further study. In addition, the potential increase in charge due to higher APTES concentrations may have been limited by the availability of −OH groups, indicating the need for further investigation into the relationship between these factors and the formation of siloxane bonds.

Despite these limitations, cationic coatings have proven to be a promising strategy for improving the performance of dental and biomedical implants. These findings emphasize the importance of using a safe APTES concentration—minimizing adverse effects while maintaining infection prevention. Additionally, the results suggest that this treatment can support the osseointegration process, a highly desirable characteristics for healthcare professionals and their patients. For future clinical implementation, deeper insights are needed into how APTES-treated surfaces interact with host tissues and diverse bacterial communities. Further studies on this topic will provide useful information to expand its clinical applicability.

## 4 Conclusions

A series of experiments was conducted to produce a multifunctional cationic coating using different concentrations of silane (APTES). Based on the results, we successfully developed a bioactive surface using PEO, which enhanced properties such as morphology, chemical composition and the production of −OH functional groups. The SEM micrographs confirmed an optimized external design, making the surface suitable for implant applications, with characteristics similar to bone structure. This treatment also incorporated Ca and P, as identified by EDS, while XPS analysis revealed the presence of protonated amines, indicating an increase in surface charge. The crystalline oxide phases formed were identified as hydroxyapatite, anatase, and rutile, further enriching the surface. After the silanization process, the different concentrations of APTES did not influence the variation in surface charge, as confirmed by the confirmed by the Zeta potential analysis. The increase in silane led to the saturation of hydroxyl groups, as the molecules require the formation of siloxane bonds (Si–O) to increase surface charge. The roughness obtained in the experimental groups was comparable to that of commercial implants. Moreover, the chemical composition and roughness contributed to surface polarization, resulting in hydrophobic surfaces with contact angles exceeding 138°. Microbiological tests showed that the cationic surfaces were effective in reducing initial bacterial adhesion by up to 65%, maintaining lower adhesion values during biofilm formation, in addition to exhibiting higher amounts of dead cells at both time points. The durability of the cationic coatings was assessed, and it was observed that after long periods of 28 days, the morphology did not undergo significant changes, with the chemical elements necessary for functionality, as well as the presence Ti–O–Si bonds, being maintained. Furthermore, surfaces treated by PEO demonstrated improved tribological behavior, exhibiting smaller wear tracks and lower mass loss, and even after mechanical contact, the predominant elements necessary for cationic surfaces could still be identified. These findings indicate that the cationic coatings remain stable and functional over time, preserving their essential chemical and mechanical properties. These results highlight those electrostatic interactions and the surface characteristics of the treatment may play a key role in preventing the development of peri-implant pathologies. Additionally, the biological findings revealed that the presence of cations aids in metabolization and cell viability; however, concentrations of APTES higher than 83.8 mM exacerbated the hydrolysis reaction, negatively impacting cell proliferation. Finally, we emphasize that the developed surface meets essential criteria for titanium implant applications, offering a promising combination of antimicrobial, cytocompatible, and bioactive properties. These results suggest a future where implants with cationic treatments not only significantly reduce the risk of infectious complications but also enhance osseointegration, representing a major advancement in implant rehabilitation.

## References

[1] Nicholson J. W. (2020). Titanium
Alloys for Dental Implants: A Review. Prosthesis.

[2] Yang J., Liu C., Sun H., Liu Y., Liu Z., Zhang D., Zhao G., Wang Q., Yang D. (2022). The Progress in Titanium
Alloys Used as Biomedical Implants: From the View of Reactive Oxygen
Species. Front. Bioeng. Biotechnol..

[3] Moraschini V., Poubel L. A. da C., Ferreira V. F., Barboza E. dos S. P. (2015). Evaluation
of Survival and Success Rates of Dental Implants Reported in Longitudinal
Studies with a Follow-up Period of at Least 10 Years: A Systematic
Review. Int. J. Oral Maxillofac. Surg..

[4] Duong H., Roccuzzo A., Stähli A., Salvi G. E., Lang N. P., Sculean A. (2022). Oral Health-related
Quality of Life of Patients Rehabilitated
with Fixed and Removable Implant-supported Dental Prostheses. Periodontol. 2000.

[5] Kurtz S. M., Lau E., Watson H., Schmier J. K., Parvizi J. (2012). Economic Burden of
Periprosthetic Joint Infection in the United States. J. Arthroplasty.

[6] Pirc M., Gadzo N., Balmer M., Naenni N., Jung R. E., Thoma D. S. (2025). Maintenance Costs, Time, and Efforts
Following Implant
Therapy With Fixed Restorations Over an Observation Period of 10 Years:
A Randomized Controlled Clinical Trial. Clin.
Implant Dent. Relat. Res..

[7] Orishko A., Imber J., Roccuzzo A., Stähli A., Salvi G. E. (2024). Tooth- and Implant-related Prognostic
Factors in Treatment
Planning. Periodontol. 2000.

[8] Daubert D. M., Weinstein B. F. (2019). Biofilm
as a Risk Factor in Implant Treatment. Periodontol.
2000.

[9] Heitz-Mayfield L. J. A., Salvi G. E. (2018). Peri-implant Mucositis. J. Clin.
Periodontol..

[10] Schwarz F., Alcoforado G., Guerrero A., Jönsson D., Klinge B., Lang N., Mattheos N., Mertens B., Pitta J., Ramanauskaite A., Sayardoust S., Sanz-Martin I., Stavropoulos A., Heitz-Mayfield L. (2021). Peri-implantitis:
Summary and Consensus Statements of Group 3. The 6th EAO Consensus
Conference 2021. Clin. Oral Implants Res..

[11] Dini C., Yamashita K. M., Sacramento C. M., Borges M. H. R., Takeda T. T. S., Silva J. P. dos S., Nagay B. E., Costa R. C., da Cruz N. C., Rangel E. C., Ruiz K. G. S., Barão V. A. R. (2025). Tailoring
Magnesium-Doped Coatings for Improving Surface and Biological Properties
of Titanium-Based Dental Implants. Colloids
Surf., B.

[12] Silva J. P. dos S., Costa R. C., Nagay B. E., Borges M. H. R., Sacramento C. M., da Cruz N. C., Rangel E. C., Fortulan C. A., da Silva J. H. D., Ruiz K. G. S., Barão V. A. R. (2023). Boosting
Titanium Surfaces with Positive
Charges: Newly Developed Cationic Coating Combines Anticorrosive and
Bactericidal Properties for Implant Application. ACS Biomater. Sci. Eng..

[13] Malheiros S. S., Borges M. H. R., Rangel E. C., Fortulan C. A., da Cruz N. C., Barao V. A. R., Nagay B. E. (2025). Zinc-Doped
Antibacterial Coating
as a Single Approach to Unlock Multifunctional and Highly Resistant
Titanium Implant Surfaces. ACS Appl. Mater.
Interfaces.

[14] Shen J., Gao P., Han S., Kao R. Y. T., Wu S., Liu X., Qian S., Chu P. K., Cheung K. M. C., Yeung K. W. K. (2020). A Tailored
Positively-Charged Hydrophobic Surface Reduces the Risk of Implant
Associated Infections. Acta Biomater..

[15] Somasundaram S. (2018). Silane Coatings
of Metallic Biomaterials for Biomedical Implants: A Preliminary Review. J. Biomed. Mater. Res. Part B Appl. Biomater..

[16] Dupraz A. M. P., Meer S. A. T. v. d., De Wijn J. R., Goedemoed J. H. (1996). Biocompatibility
Screening of Silane-Treated Hydroxyapatite Powders, for Use as Filler
in Resorbable Composites. J. Mater. Sci. Mater.
Med..

[17] Ahangaran F., Navarchian A. H. (2020). Recent
Advances in Chemical Surface Modification of
Metal Oxide Nanoparticles with Silane Coupling Agents: A Review. Adv. Colloid Interface Sci..

[18] Ara C., Jabeen S., Afshan G., Farooq A., Akram M. S., Asmatullah, Islam A., Ziafat S., Nawaz B., Khan R. U. (2022). Angiogenic Potential
and Wound Healing Efficacy of Chitosan Derived Hydrogels at Varied
Concentrations of APTES in Chick and Mouse Models. Int. J. Biol. Macromol..

[19] Chuang F., Tsen W., Shu Y. (2004). The Effect
of Different Siloxane
Chain-Extenders on the Thermal Degradation and Stability of Segmented
Polyurethanes. Polym. Degrad. Stab..

[20] Costa R. C., Nagay B. E., Dini C., Borges M. H. R., Miranda L. F. B., Cordeiro J. M., Souza J. G. S., Sukotjo C., Cruz N. C., Barão V. A. R. (2023). The
Race for the Optimal Antimicrobial Surface: Perspectives
and Challenges Related to Plasma Electrolytic Oxidation Coating for
Titanium-Based Implants. Adv. Colloid Interface
Sci..

[21] Andrade C. S., Borges M. H. R., Silva J. P., Malheiros S., Sacramento C., Ruiz K. G. S., da
Cruz N. C., Rangel E. C., Fortulan C., Figueiredo L., Nagay B. E., Souza J. G. S., Barão V. A. R. (2025). Micro-Arc
Driven Porous ZrO2 Coating
for Tailoring Surface Properties of Titanium for Dental Implants Application. Colloids Surfaces B Biointerfaces.

[22] Marques I. da S. V., Barão V. A. R., Cruz N. C. da, Yuan J. C.-C., Mesquita M. F., Ricomini-Filho A. P., Sukotjo C., Mathew M. T. (2015). Electrochemical
Behavior of Bioactive Coatings on Cp-Ti Surface for Dental Application. Corros. Sci..

[23] Borges M. H. R., Nagay B. E., Costa R. C., Sacramento C. M., Ruiz K. G., Landers R., van den Beucken J. J. J. P., Fortulan C. A., Rangel E. C., da Cruz N. C., Barão V. A. R. (2022). A Tattoo-Inspired
Electrosynthesized Polypyrrole Film: Crossing the Line toward a Highly
Adherent Film for Biomedical Implant Applications. Mater. Today Chem..

[24] Wu J., Zhou L., Ding X., Gao Y., Liu X. (2015). Biological
Effect of Ultraviolet Photocatalysis on Nanoscale Titanium with a
Focus on Physicochemical Mechanism. Langmuir.

[25] Moreno D., Buxadera-Palomero J., Ginebra M.-P., Manero J.-M., Martin-Gómez H., Mas-Moruno C., Rodríguez D. (2023). Comparison of the Antibacterial Effect
of Silver Nanoparticles and a Multifunctional Antimicrobial Peptide
on Titanium Surface. Int. J. Mol. Sci..

[26] Li L., Sun W., Yu J., Lei W., Zeng H., Shi B. (2022). Effects of
Titanium Dioxide Microparticles and Nanoparticles on Cytoskeletal
Organization, Cell Adhesion, Migration, and Proliferation in Human
Gingival Fibroblasts in the Presence of Lipopolysaccharide. J. Periodontal Res..

[27] Nikoomanzari E., Karbasi M., Melo W. C. M. A., Moris H., Babaei K., Giannakis S., Fattah-alhosseini A. (2022). Impressive Strides in Antibacterial
Performance Amelioration of Ti-Based Implants via Plasma Electrolytic
Oxidation (PEO): A Review of the Recent Advancements. Chem. Eng. J..

[28] Marques I. da S. V., da Cruz N. C., Landers R., Yuan J. C.-C., Mesquita M. F., Sukotjo C., Mathew M. T., Barão V. A. R. (2015). Incorporation
of Ca, P, and Si on Bioactive Coatings Produced by Plasma Electrolytic
Oxidation: The Role of Electrolyte Concentration and Treatment Duration. Biointerphases.

[29] Santos-Coquillat A., Martínez-Campos E., Mohedano M., Martínez-Corriá R., Ramos V., Arrabal R., Matykina E. (2018). In Vitro and in Vivo
Evaluation of PEO-Modified Titanium for Bone Implant Applications. Surf. Coat. Technol..

[30] Fattah-alhosseini A., Molaei M., Attarzadeh N., Babaei K., Attarzadeh F. (2020). On the Enhanced
Antibacterial Activity of Plasma Electrolytic Oxidation (PEO) Coatings
That Incorporate Particles: A Review. Ceram.
Int..

[31] Loscutoff P. W., Zhou H., Clendenning S. B., Bent S. F. (2010). Formation of Organic
Nanoscale Laminates and Blends by Molecular Layer Deposition. ACS Nano.

[32] Xia Y., Zhao X.-M., Whitesides G. M. (1996). Pattern Transfer: Self-Assembled
Monolayers as Ultrathin Resists. Microelectron.
Eng..

[33] Howarter J. A., Youngblood J. P. (2006). Optimization of Silica Silanization
by 3-Aminopropyltriethoxysilane. Langmuir.

[34] Ranjit E., Hamlet S., Love R. M. (2023). Keratin
Coated Titanium as an Aid
to Osseointegration: Physicochemical and Mechanical Properties. Surf. Coat. Technol..

[35] Kharbanda O. P., Sharan J., Koul V., Dinda A. K., Mishra M., Gupta G., Singh M. P. (2017). Tethering
of 3-Aminopropyltriethoxy
Silane Films on Medical Grade V Titanium Alloy Surface through Self-Assembled
Monolayers (SAMs) for Biomedical Applications. Appl. Surf. Sci..

[36] Miranda A., Martínez L., De Beule P. A. A. (2020). Facile Synthesis
of an Aminopropylsilane
Layer on Si/SiO2 Substrates Using Ethanol as APTES Solvent. MethodsX.

[37] de Carvalho, R. ; Araújo, M. G. da F. Hidroxiapatitas Organofuncionalizadas Como Sistemas Para Biorremediação de Corante Aniônico, Universidade Federal da Paraíba; João Pessoa, 2016.

[38] Matinlinna J. P., Areva S., Lassila L. V. J., Vallittu P. K. (2004). Characterization
of Siloxane Films on Titanium Substrate Derived from Three Aminosilanes. Surf. Interface Anal..

[39] Ederer J., Janoš P., Ecorchard P., Tolasz J., Štengl V., Beneš H., Perchacz M., Pop-Georgievski O. (2017). Determination
of Amino Groups on Functionalized Graphene Oxide for Polyurethane
Nanomaterials: XPS Quantitation vs. Functional Speciation. RSC Adv..

[40] Talavera-Pech W. A., Esparza-Ruiz A., Quintana-Owen P., Vilchis-Nestor A. R., Carrera-Figueiras C., Ávila-Ortega A. (2016). Effects of Different Amounts of APTES
on Physicochemical and Structural Properties of Amino-Functionalized
MCM-41-MSNs. J. Sol-Gel Sci. Technol..

[41] Agrawal N., Munjal S., Ansari M. Z., Khare N. (2017). Superhydrophobic Palmitic
Acid Modified ZnO Nanoparticles. Ceram. Int..

[42] Lu G., Bernasek S. L., Schwartz J. (2000). Oxidation of a Polycrystalline Titanium
Surface by Oxygen and Water. Surf. Sci..

[43] Tan G., Zhang L., Ning C., Liu X., Liao J. (2011). Preparation
and Characterization of APTES Films on Modification Titanium by SAMs. Thin Solid Films.

[44] Ruiz J., St-Georges-Robillard A., Thérésy C., Lerouge S., Wertheimer M. R. (2010). Fabrication
and Characterisation of Amine-Rich Organic
Thin Films: Focus on Stability. Plasma Process.
Polym..

[45] Bai Y., Li Z., Cheng B., Zhang M., Su K. (2017). Higher UV-Shielding
Ability and Lower Photocatalytic Activity of TiO _2_ @SiO _2_ /APTES and Its Excellent Performance in Enhancing the Photostability
of Poly­(p-Phenylene Sulfide). RSC Adv..

[46] Liu Y., Bai Z., Lin G., Wang L., Xu X., He L., Liu X. (2022). Covalent Cross-Linking
Mediated TA-APTES NPs to Construct a High-Efficiency
GO Composite Membrane for Dye/Salt Separation. Appl. Surf. Sci..

[47] Wojciechowski J., Kolanowski Ł., Graś M., Szubert K., Bund A., Fic K., Lota G. (2021). Anti–Corrosive
Siloxane Coatings for Improved
Long–Term Performance of Supercapacitors with an Aqueous Electrolyte. Electrochim. Acta.

[48] Díaz I., Chico B., de la Fuente D., Simancas J., Vega J. M., Morcillo M. (2010). Corrosion Resistance
of New Epoxy–Siloxane Hybrid
Coatings. A Laboratory Study. Prog. Org. Coatings.

[49] Beline T., Marques I. da S. V., Matos A. O., Ogawa E. S., Ricomini-Filho A. P., Rangel E. C., da Cruz N. C., Sukotjo C., Mathew M. T., Landers R., Consani R. L. X., Mesquita M. F., Barão V. A. R. (2016). Production
of a Biofunctional Titanium Surface Using Plasma Electrolytic Oxidation
and Glow-Discharge Plasma for Biomedical Applications. Biointerphases.

[50] Mashtalyar D. V., Imshinetskiy I. M., Nadaraia K. V., Gnedenkov A. S., Suchkov S. N., Opra D. P., Pustovalov E. V., Yu Ustinov A., Sinebryukhov S. L., Gnedenkov S. V. (2023). Effect
of TiO2 Nanoparticles on the Photocatalytic Properties of PEO Coatings
on Mg Alloy. J. Magnes. Alloy..

[51] Yang X., Chen Y., Yang F., He F.-M., Zhao S. (2009). Enhanced Initial
Adhesion of Osteoblast-like Cells on an Anatase-Structured Titania
Surface Formed by H2O2/HCl Solution and Heat Treatment. Dent. Mater..

[52] Rheima A. M., Khadom A. A., Kadhim M. M., Al-Uqaily R. A. H., Mohammed S. H. (2022). Electrochemical Synthesis of Rutile Phase Titanium
Dioxide Nanosheets for Corrosion Protection of Mild Steel in Acidic
Media. J. Bio- Tribo-Corrosion.

[53] e
Silva M. L. de M., Horta M. K. dos S., Ferreira R. D., do Nascimento M. V. B., e Oliveira R. L., Junior S. D., de Castro D. A. R. (2022). Síntese
e Caracterização Da Hidroxiapatita Por Meio de Tratamento
Alcalino Utilizando Escamas Do Aruanã (Osteoglossum Bicirrhosum). Brazilian J. Dev..

[54] Guilherme Tamarozzi Justino. Hydroxyapatite Obtained via Micro-Wave/Hydrothermal; Universidade Tecnológica Federal do Paraná: Londrina, 2016.

[55] Reis-Neta G. R. dos, Ricomini-Filho A. P., Martorano-Fernandes L., Vargas-Moreno V. F., Cury A. A. D. B., Marcello-Machado R. M. (2024). Effect
of Hydroxyapatite Nanoparticles Coating of Titanium Surface on Biofilm
Adhesion: An in Vitro Study. Arch. Oral Biol..

[56] Muráth S., Sáringer S., Somosi Z., Szilágyi I. (2018). Effect of
Ionic Compounds of Different Valences on the Stability of Titanium
Oxide Colloids. Colloids and Interfaces.

[57] Reggio C., Barberi J., Ferraris S., Spriano S. (2023). Functionalization of
Ti6Al4V Alloy with Polyphenols: The Role of the Titanium Surface Features
and the Addition of Calcium Ions on the Adsorption Mechanism. Metals (Basel)..

[58] Tamba B. I., Dondas A., Leon M., Neagu A. N., Dodi G., Stefanescu C., Tijani A. (2015). Silica Nanoparticles: Preparation,
Characterization and in Vitro/in Vivo Biodistribution Studies. Eur. J. Pharm. Sci..

[59] Sevilla P., Gil J., Aparicio C. (2017). Relevant Properties
for Immobilizing Short Peptides
on Biosurfaces. IRBM.

[60] Siqueira
Petri D. F., Wenz G., Schunk P., Schimmel T. (1999). An Improved
Method for the Assembly of Amino-Terminated Monolayers on SiO _2_ and the Vapor Deposition of Gold Layers. Langmuir.

[61] Albrektsson T., Wennerberg A. (2004). Oral Implant Surfaces: Part 1—Review Focusing
on Topographic and Chemical Properties of Different Surfaces and in
Vivo Responses to Them. Int. J. Prosthodont..

[62] Nicolas-Silvente A. I., Velasco-Ortega E., Ortiz-Garcia I., Monsalve-Guil L., Gil J., Jimenez-Guerra A. (2020). Influence of the Titanium Implant
Surface Treatment on the Surface Roughness and Chemical Composition. Materials (Basel)..

[63] Stich T., Alagboso F., Křenek T., Kovářík T., Alt V., Docheva D. (2022). Implant-bone-interface: Reviewing the Impact of Titanium
Surface Modifications on Osteogenic Processes in Vitro and in Vivo. Bioeng. Transl. Med..

[64] Souza J. C. M., Sordi M. B., Kanazawa M., Ravindran S., Henriques B., Silva F. S., Aparicio C., Cooper L. F. (2019). Nano-Scale
Modification of Titanium Implant Surfaces to Enhance Osseointegration. Acta Biomater..

[65] Romero-Serrano M., Romero-Ruiz M.-M., Herrero-Climent M., Rios-Carrasco B., Gil-Mur J. (2024). Correlation between Implant Surface
Roughness and Implant
Stability: A Systematic Review. Dent. J..

[66] Rupp F., Scheideler L., Rehbein D., Axmann D., Geis-Gerstorfer J. (2004). Roughness
Induced Dynamic Changes of Wettability of Acid Etched Titanium Implant
Modifications. Biomaterials.

[67] Lotz E. M., Olivares-Navarrete R., Berner S., Boyan B. D., Schwartz Z. (2016). Osteogenic
Response of Human MSCs and Osteoblasts to Hydrophilic and Hydrophobic
Nanostructured Titanium Implant Surfaces. J.
Biomed. Mater. Res. Part A.

[68] Vasak C., Busenlechner D., Schwarze U. Y., Leitner H. F., Munoz
Guzon F., Hefti T., Schlottig F., Gruber R. (2014). Early Bone Apposition to Hydrophilic and Hydrophobic
Titanium Implant Surfaces: A Histologic and Histomorphometric Study
in Minipigs. Clin. Oral Implants Res..

[69] Fan H., Guo Z. (2020). Bioinspired Surfaces
with Wettability: Biomolecule Adhesion Behaviors. Biomater. Sci..

[70] Uppu D. S. S. M., Samaddar S., Hoque J., Konai M. M., Krishnamoorthy P., Shome B. R., Haldar J. (2016). Side Chain
Degradable Cationic–Amphiphilic
Polymers with Tunable Hydrophobicity Show in Vivo Activity. Biomacromolecules.

[71] Marín-Pareja N., Cantini M., González-García C., Salvagni E., Salmerón-Sánchez M., Ginebra M.-P. (2015). Different Organization of Type I Collagen Immobilized
on Silanized and Nonsilanized Titanium Surfaces Affects Fibroblast
Adhesion and Fibronectin Secretion. ACS Appl.
Mater. Interfaces.

[72] Souza J. G. S., Bertolini M., Costa R. C., Cordeiro J. M., Nagay B. E., de Almeida A. B., Retamal-Valdes B., Nociti F. H., Feres M., Rangel E. C., Barão V. A. R. (2020). Targeting Pathogenic Biofilms: Newly
Developed Superhydrophobic Coating Favors a Host-Compatible Microbial
Profile on the Titanium Surface. ACS Appl. Mater.
Interfaces.

[73] Barberi J., Spriano S. (2021). Titanium and Protein Adsorption: An Overview of Mechanisms
and Effects of Surface Features. Materials (Basel)..

[74] Yang D.-Q., Meunier M., Sacher E. (2005). Photoacoustic
Fourier Transform Infrared
Spectroscopy of Nanoporous SiOx/Si Thin Films with Varying Porosities. J. Appl. Phys..

[75] Arkles, B. ; Hydrophobicity, Hydrophilicity and Silane Surface Modification. Gelest, Inc, 2011.

[76] Wang D., Bierwagen G. P. (2009). Sol–Gel Coatings on Metals for Corrosion Protection. Prog. Org. Coatings.

[77] de
Oliveira L., Rigotti R., Dias Corpa Tardelli J., Cândido dos Reis A. (2023). Influence of Antibacterial Surface
Treatment on Dental Implants on Cell Viability: A Systematic Review. Heliyon.

[78] Frieri M., Kumar K., Boutin A. (2017). Antibiotic Resistance. J. Infect. Public Health.

[79] Arciola C. R., Campoccia D., Montanaro L. (2018). Implant Infections: Adhesion, Biofilm
Formation and Immune Evasion. Nat. Rev. Microbiol..

[80] Zhang X., Wang L., Levänen E. (2013). Superhydrophobic Surfaces for the
Reduction of Bacterial Adhesion. RSC Adv..

[81] Tian X., Xue R., Yang F., Yin L., Luan S., Tang H. (2021). Single-Chain
Nanoparticle-Based Coatings with Improved Bactericidal Activity and
Antifouling Properties. Biomacromolecules.

[82] Steinbach G., Crisan C., Ng S. L., Hammer B. K., Yunker P. J. (2020). Accumulation
of Dead Cells from Contact Killing Facilitates Coexistence in Bacterial
Biofilms. J. R. Soc. Interface.

[83] Garg P., Mazur M. M., Buck A. C., Wandtke M. E., Liu J., Ebraheim N. A. (2017). Prospective Review
of Mesenchymal Stem Cells Differentiation
into Osteoblasts. Orthop. Surg..

[84] Iglic A., Gongadze, Kabaso D., Bauer, Schmuki P., Slivnik, van
Rienen U. (2011). Adhesion of Osteoblasts to a Nanorough Titanium Implant Surface. Int. J. Nanomedicine.

[85] Kabaso D., Gongadze E., Perutková Š., Matschegewski C., Kralj-Iglič V., Beck U., van Rienen U., Iglič A. (2011). Mechanics
and Electrostatics of the Interactions between
Osteoblasts and Titanium Surface. Comput. Methods
Biomech. Biomed. Engin..

[86] Tavelli L., McGuire M. K., Zucchelli G., Rasperini G., Feinberg S. E., Wang H., Giannobile W. V. (2020). Extracellular
Matrix-based Scaffolding Technologies for Periodontal and Peri-implant
Soft Tissue Regeneration. J. Periodontol..

[87] Bini R. A., Marques R. F. C., Santos F. J., Chaker J. A., Jafelicci M. (2012). Synthesis
and Functionalization of Magnetite Nanoparticles with Different Amino-Functional
Alkoxysilanes. J. Magn. Magn. Mater..

[88] Judson R., Houck K., Martin M., Richard A. M., Knudsen T. B., Shah I., Little S., Wambaugh J., Woodrow
Setzer R., Kothya P., Phuong J., Filer D., Smith D., Reif D., Rotroff D., Kleinstreuer N., Sipes N., Xia M., Huang R., Crofton K., Thomas R. S. (2016). Editor’s Highlight: Analysis of the Effects
of Cell Stress and Cytotoxicity on In Vitro Assay Activity Across
a Diverse Chemical and Assay Space. Toxicol.
Sci..

[89] Goldstein, J. I. ; Newbury, D. E. ; Michael, J. R. ; Ritchie, N. W. M. ; Scott, J. H. J. ; Joy, D. C. Scanning Electron Microscopy and X-Ray Microanalysis; Springer New York: New York, NY, 2018. 10.1007/978-1-4939-6676-9.

[90] Vandrovcova M., Tolde Z., Vanek P., Nehasil V., Doubková M., Trávníčková M., Drahokoupil J., Buixaderas E., Borodavka F., Novakova J., Bacakova L. (2021). Beta-Titanium
Alloy Covered by Ferroelectric Coating–Physicochemical Properties
and Human Osteoblast-Like Cell Response. Coatings.

[91] Ueda E., Levkin P. A. (2013). Emerging Applications
of Superhydrophilic-Superhydrophobic
Micropatterns. Adv. Mater..

